# Identification of Clinically Distinct Clusters in Patients With Severe COPD Using Circulating Blood Cell Population Parameters

**DOI:** 10.1002/resp.70146

**Published:** 2025-10-19

**Authors:** Pauline J. M. Kuks, Jorine E. Hartman, Else A. M. D. ter Haar, L. Joost van Pelt, Dirk‐Jan Slebos, Maarten van den Berge, Simon D. Pouwels

**Affiliations:** ^1^ Department of Pulmonary Diseases University of Groningen, University Medical Center Groningen Groningen the Netherlands; ^2^ Groningen Research Institute for Asthma and COPD, University of Groningen, University Medical Center Groningen Groningen the Netherlands; ^3^ Department of Laboratory Medicine University of Groningen, University Medical Center Groningen Groningen the Netherlands; ^4^ Department of Pathology and Medical Biology University of Groningen, University Medical Center Groningen Groningen the Netherlands

**Keywords:** biomarkers, cell population data, cluster analysis, COPD, inflammatory activity

## Abstract

**Background and Objective:**

Peripheral blood cell counts are useful biomarkers in COPD, but may not fully reflect disease activity. The Sysmex XN‐Series haematology analyser offers advanced measurements of immune cell populations, providing information about the number and activation status of peripheral blood cells. We hypothesized that assessing immune cell activation status, in addition to cell counts, could provide complementary insights into the clinical heterogeneity of severe COPD.

**Methods:**

For this study, 499 extensively characterised patients with severe COPD were included from the Groningen Severe COPD cohort. A total of 24 Sysmex‐derived systemic blood parameters were selected for analysis. Clustering of blood cell population data was performed using Self‐Organising Maps.

**Results:**

The cell population parameters showed various associations with clinical characteristics, such as emphysema severity and lung function. Four clusters were identified based on their inflammatory profiles, each showing distinct clinical characteristics: the ‘normal cell counts, resting pattern’ cluster (*n* = 156) showed high emphysema severity scores and RV/TLC ratios; the ‘normal cell counts, activated pattern’ cluster (*n* = 241) was associated with few exacerbations; the ‘elevated cell counts, activated pattern*’* cluster (*n* = 97) displayed high inflammatory cell counts and activity along with high exacerbation rates; and the small ‘*low‐eosinophilic’* cluster (*n* = 5) was characterised by inactive circulating eosinophils.

**Conclusion:**

Cell population data can be used to identify distinct inflammatory profiles with clinical relevance in severe COPD. Cell population data provide information beyond absolute cell counts, supporting the added value of including activation markers in COPD phenotyping.

**Trial Registration:**

NCT04023409 at clinicaltrials.gov

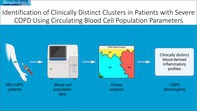

## Graphical abstract

This study investigated whether blood cell population data, in addition to cell counts, can identify clinically relevant COPD subgroups. Four distinct inflammatory profiles were identified with distinct clinical characteristics. Cell population data have the potential to provide additional insights beyond inflammatory cell counts and improve patient classification and clinical decision‐making.

**See related** editorial

## 1 Introduction

Chronic obstructive pulmonary disease (COPD) is a heterogeneous and progressive lung disorder, characterised by persistent respiratory symptoms and airflow limitation [1]. Inflammation in COPD plays a key role in driving structural and functional lung changes and has also been linked to a range of comorbidities including cardiovascular disease, osteoporosis, and muscle wasting [2, 3]. Inflammatory markers in peripheral blood have emerged as potential biomarkers for disease severity and treatment response [4, 5]. In patients with COPD, elevated peripheral blood neutrophil counts are associated with greater disease severity, as evidenced by a higher GOLD classification, more frequent and more severe exacerbations, and an increased mortality risk [6, 7]. Elevated blood eosinophil counts are associated with exacerbations, FEV_1_ decline, and a greater response to corticosteroid therapy and may serve as a biomarker for identifying patients who could benefit from such treatment [8, 9, 10].

However, absolute numbers of circulating inflammatory cells do not fully explain disease activity. Inflammation‐driven tissue damage is not only determined by the number of circulating immune cells, but also by their activation status, as reflected by changes in granularity, nucleic acid content, and cell size [11, 12]. Moreover, studying single blood parameters may overlook interactions between different cellular components of the immune system. Combining multiple blood markers, including immune cell counts, activation markers, platelet indices, and red blood cell characteristics, into a composite inflammatory profile could improve the ability to capture disease heterogeneity and predict outcomes.

The Sysmex XN‐Series haematology analyser (Sysmex Corp., Kobe, Japan) is a single colour flow cytometer that diagnostic laboratories can use to report blood cell counts and white blood cell differentials [13]. Primarily, the instrument is designed to count different subtypes of blood cells (red blood cells, white blood cells and their common subtypes, and platelets). The analyser software clusters the cells based on their forward scatter (FCS), side scatter (SSC), and fluorescence (SFL) patterns. An important feature of the analyser is the recognition of aberrant cell differentiation in haematological disease (e.g., leukaemia). The cell counts are reportable parameters, while cell cluster parameters are research use only parameters that are not commonly reported. These parameters are referred to as cell population data (CPD).

The CPD may reflect cell function and activation. The side‐scattered light distribution reflects cell granularity and internal structural features, such as the distribution and density of organelles and granules [14, 15, 16, 17]. The fluorescent light intensity reflects the nucleic acid content, which offers insights into metabolic activity and cell maturation, important both for identifying functional changes related to cell differentiation or activation [15, 16, 17]. The forward scattered light is correlated with cellular size, a parameter related to different stages of cell development and cell activation, which could help to detect subtle changes in cell function during immune responses or disease progression [15, 16, 17]. In addition to their use in blood cell differentiation, these parameters enable the detection of specific immune cell populations, such as antibody‐synthesising lymphocytes, reactive lymphocytes, and immature granulocytes, as well as information about the activation status of neutrophils, eosinophils, monocytes, and lymphocytes.

The CPD has been studied as potential biomarkers in conditions such as sepsis [18, 19]. Their application in COPD remains unexplored. In the current study, we evaluated the CPD in 499 COPD patients from the Groningen Severe COPD cohort (GSCC). The aim of this study was to evaluate whether peripheral blood CPD can identify subgroups of patients with severe COPD with distinct inflammatory characteristics, and whether these subgroups differ in terms of disease severity, comorbidities, and structural lung abnormalities.

## 2 Methods

### 2.1 Study Design and Participants

This study utilized data obtained from a subgroup of subjects of the Groningen Severe COPD Cohort (GSCC), an observational cross‐sectional cohort that included 1030 patients with severe COPD referred to the University Medical Center Groningen in The Netherlands for bronchoscopic lung volume reduction (BLVR) treatment between August 2019 and July 2024. Blood was collected prior to BLVR treatment in EDTA‐anticoagulation tubes, transported to the lab immediately, and analysed within 30 min upon arrival at the lab. Blood was always drawn between 11:00 AM and 1:00 PM. For the current analysis, patients were selected of which the blood cell counts and CPD data were available. Patients with a C‐reactive protein (CRP) level above 20 mg/L were excluded in order to exclude patients with an acute infection. Additionally, patients that used any dosage of oral prednisone were excluded as this could suppress inflammatory markers and thus interfere with accurate assessment of systemic inflammation.

### 2.2 Selection of Parameters

A total of 24 systemic blood parameters were selected for analysis (Table 1). The 24 systemic blood parameters included absolute cell counts (e.g., neutrophils, eosinophils, lymphocytes, and monocytes), specific lymphocyte populations (antibody‐synthesising lymphocytes, reactive lymphocytes), immature granulocytes, CPD based on forward scatter, side scatter, and fluorescence, and markers related to erythrocytes and platelets.

**TABLE 1:** Cell population parameters with their reference intervals.

XN parameter	Explanation	Reference intervals
AS‐LYMP	Antibody‐synthesising lymphocytes	0.00–0.00 * 10^9^/L
EO	Eosinophils (absolute count)	0.05–0.53 * 10^9^/L
EO‐X	Side scattered light distribution of the eosinophil population	182–203 channel
EO‐Y	Fluorescent light distribution of the eosinophil population	33.4–38.7 channel
EO‐Z	Forward scattered light distribution of the eosinophil population	97–113 channel
HGB	Haemoglobin concentration	Males: 134–170 g/L Females: 118–152 g/L
IG	Immature granulocytes (absolute count of metamyelocytes, myelocytes and promyelocytes)	0.01–0.07 * 10^9^/L
LYMPH	Lymphocytes (absolute count)	1.1–3.3 * 10^9^/L
LY‐X	Side scattered light distribution of the lymphocyte population	74.6–80.8 channel
LY‐Y	Fluorescent light distribution of the lymphocyte population	63.5–74.2 channel
LY‐Z	Forward scattered light distribution of the lymphocyte population	58.5–63.2 channel
MacroR	Macrocytic red blood cells	3.1%–4.5%
MicroR	Microcytic red blood cells	0.3%–3.3%
MONO	Monocytes (absolute count)	0.3–0.8 * 10^9^/L
MO‐X	Side scattered light distribution of the monocyte population	115–121 channel
MO‐Y	Fluorescent scattered light of the monocyte population	99–118 channel
MO‐Z	Forward scattered light of the monocyte population	64.2–72.4 channel
NEUT	Neutrophils (absolute count)	1.6–5.8 * 10^9^/L
NEUT‐GI	Neutrophil granularity intensity (formerly NEUT‐SSC or NEUT‐X)	142.4–157.0 Scatter intensity
NEUT‐RI	Neutrophil reactivity intensity (formerly NEUT‐SFL or NEUT‐Y)	42.0–50.6 Fluorescence intensity
NE‐Z	Forward scattered light distribution of the neutrophil population (formerly NE‐FSC)	85.5–97.4 channel
P‐LCR	Platelet large cell ratio	19.3%–47.1%
PLT	Platelet count	167–377 * 10^9^/Litre
RE‐LYMP	Reactive lymphocytes	0.03–0.17 * 10^9^/Litre

### 2.3 Clinical Variables

Clinical characteristics included demographics, use of inhaled corticosteroids, exacerbation history, symptom burden assessed by validated questionnaires (COPD Assessment Test [CAT], Clinical COPD Questionnaire [CCQ], Medical Research Council dyspnoea scale [mMRC], and St. George's Respiratory Questionnaire [SGRQ]), lung function parameters, computed tomography (CT)‐derived imaging markers, and the presence of comorbidities. Comorbidities were determined based on a self‐administered questionnaire and validated through medical records; definitions were previously published by ter Haar et al. [20].

### 2.4 Analyses

Deviations from the normal range for the selected CPD were assessed using the reference values established by van Pelt et al. [21] These reference intervals are based on 15,803 healthy individuals in the Dutch Lifelines study. Cluster analyses were performed using the 24 cell population variables to categorise patients based on their distinct blood profile characteristics. Clustering was conducted with Viscovery‐SOMine (v7.2; Viscovery Software, Vienna, Austria) using the Self‐Organising Maps (SOM)–Ward method, an unsupervised hierarchical clustering approach that groups patients into homogeneous clusters based on the similarities in their haematological features. Afterwards, differences in clinical characteristics between clusters were assessed.

Statistical analyses were conducted using RStudio (version 4.3.3; R Foundation for Statistical Computing, Vienna, Austria). A *p*‐value less than 0.05 was considered statistically significant. Tables were created using the “Tableone” package (version 0.13.0), and figures were generated with the “ggplot2” package (version 3.3.1). Clinical differences between the clusters were tested using appropriate statistical tests: numerical variables were compared across multiple groups using an Analysis of Variance (ANOVA) with post hoc Bonferroni correction, or, in case of non‐normality, using the Kruskal–Wallis test followed by Dunn's test with Bonferroni correction. For categorical variables, a chi‐squared test was used to examine the differences in variables between the groups. Pearson's correlation test was used to assess relationships between the haematological parameters and continuous clinical variables.

## 3 Results

**FIGURE 1:**
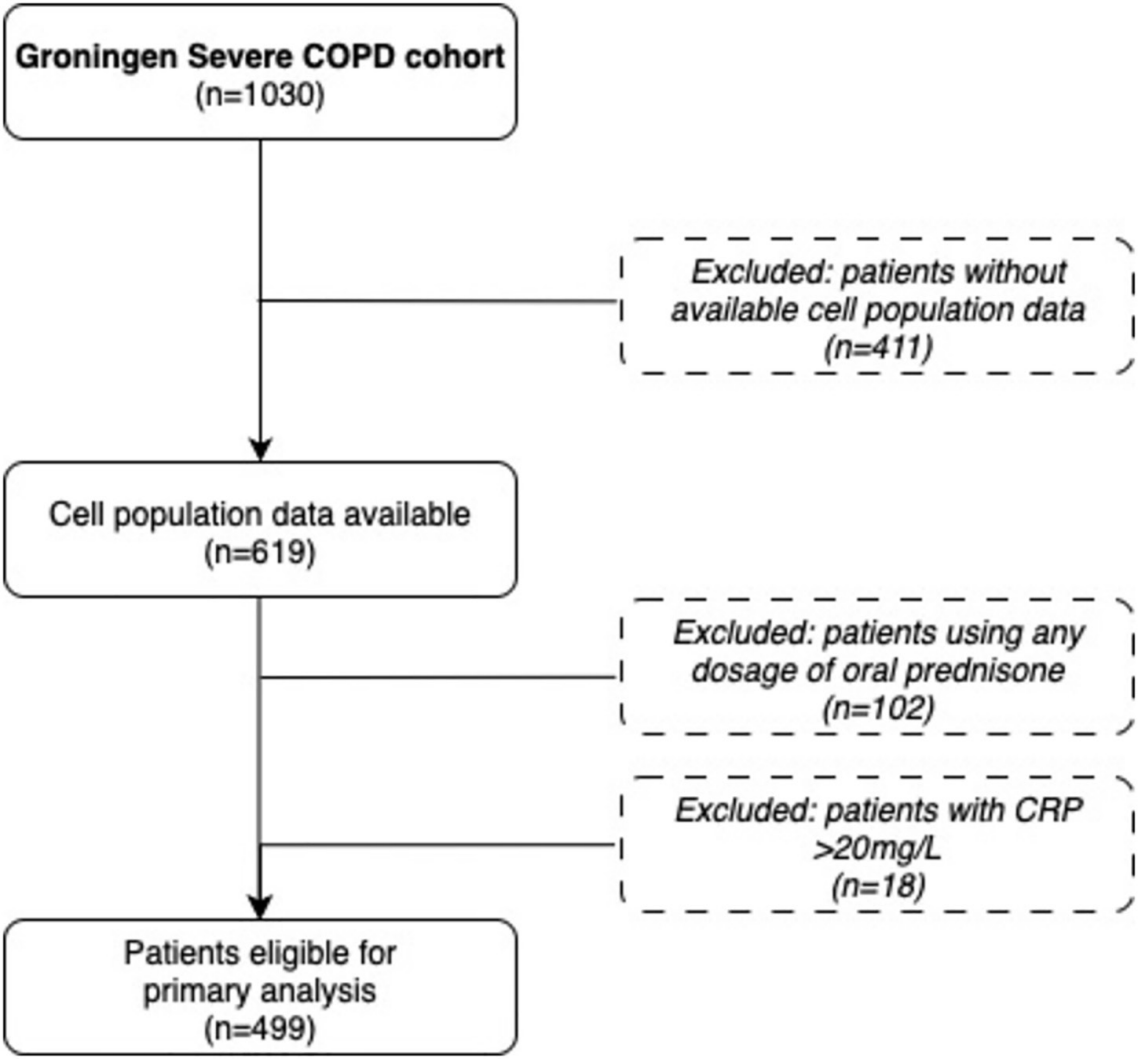
Flowchart of patient selection for the primary analysis. From the Groningen Severe COPD cohort (*n* = 1030), patients were excluded if cell population data were unavailable (*n* = 411), if they were using any dosage of oral prednisone (*n* = 102), or if C‐reactive protein (CRP) levels exceeded 20 mg/L (*n* = 18).

A total of 499 patients with COPD (69% female, mean ± SD age 61.7 ± 7.43 years, 2% current smokers, forced expiratory volume in 1 s (FEV_1_) 29.0% ± 9.79% predicted) were included in this analysis (Figure 1) (Table S1).

### 3.1 Prevalence of Deviations From Reference Interval of the Cell Population Data (CPD)

**FIGURE 2:**
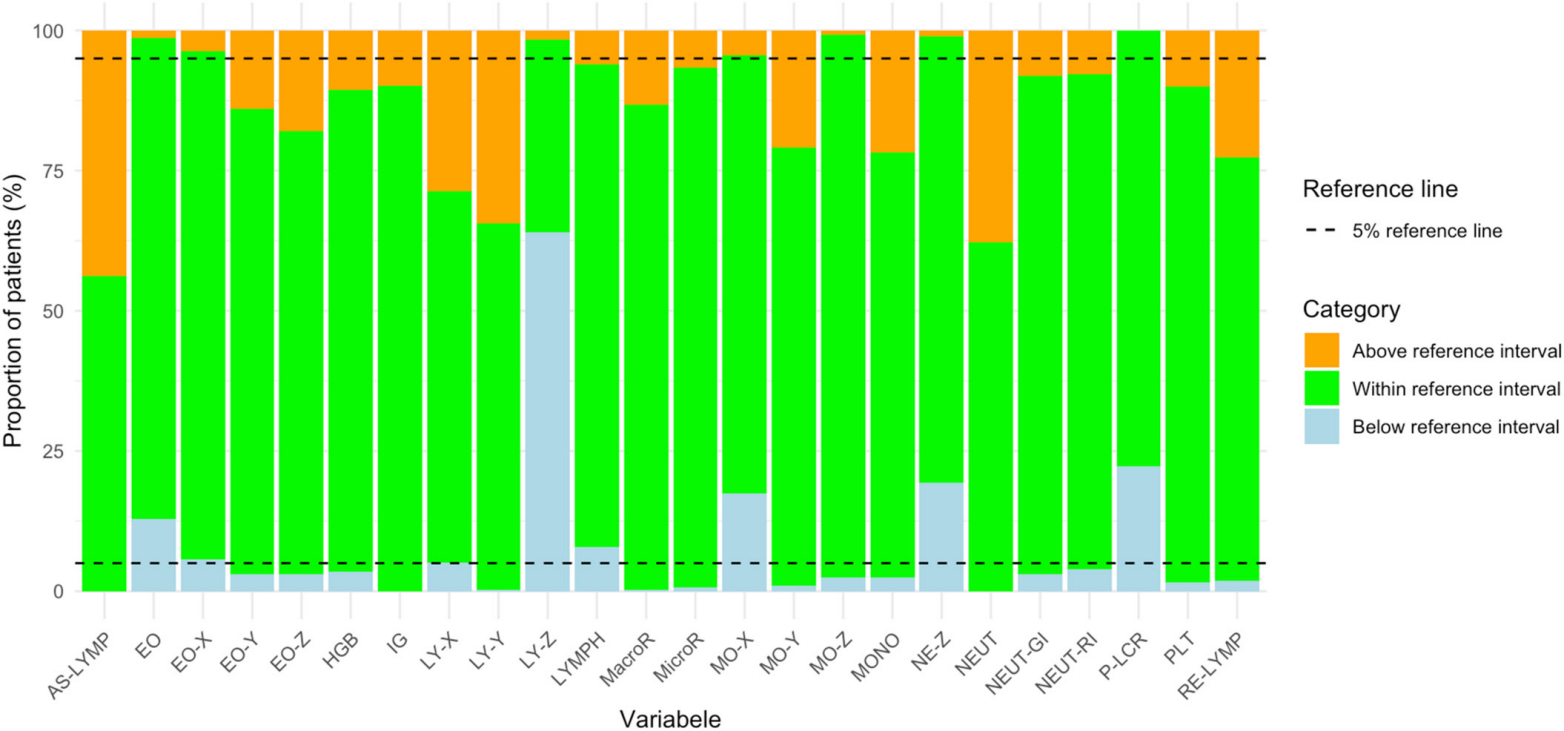
Proportion of patients with values above, within and below the reference interval for each cell population parameter. The green bars indicating the proportion of patients within the reference interval, orange above, and blue below. The dashed line represents the 5% thresholds for out‐of‐range values.

Significant deviations of at least 5% of the patients above or below the reference value were found for all variables except for the size of the monocytes (MO‐Z). Both the number of lymphocytes (LYMPH) and the side scatter of lymphocytes (LY‐X) exhibited deviations in both directions, with at least 5% of patients above and below the reference value (Figure 2).

### 3.2 Associations Between Cell Population Parameters and Clinical Characteristics

Clinical characteristics were compared between patients with CPD outside the reference interval and those within the normal range. A summary of clinical differences is provided in Table S2; full univariate results are presented in Tables S3.1–S3.26. Briefly, patients with elevated immature granulocyte (IG) counts had more frequent exacerbations in the previous year (median 2.0 vs. 1.0, *p* < 0.001). A higher proportion of macrocytic red blood cells (MacroR) was observed in patients with a history of myocardial infarction (14.5% vs. 4.7%, *p* = 0.010), while elevated microcytic red blood cell counts (MicroR) were associated with higher CAT scores (24.5 vs. 21.7, *p* = 0.010). Patients with decreased monocyte side scattering (MO‐X) showed less emphysema destruction (37.4% vs. 34.8%, *p* = 0.016), whereas increased monocyte fluorescence (MO‐Y) was linked to a higher prevalence of pulmonary arterial hypertension (14% vs. 6%, *p* = 0.021). Patients with neutrophil counts (NEUT) above the reference interval showed lower FEV_1_%predicted values (27% vs. 30%, *p* = 0.003), and those with elevated neutrophil reactivity intensities (NEUT‐RI) showed more extensive emphysema destruction (38.2% vs. 34.8%, *p* = 0.029).

**FIGURE 3:**
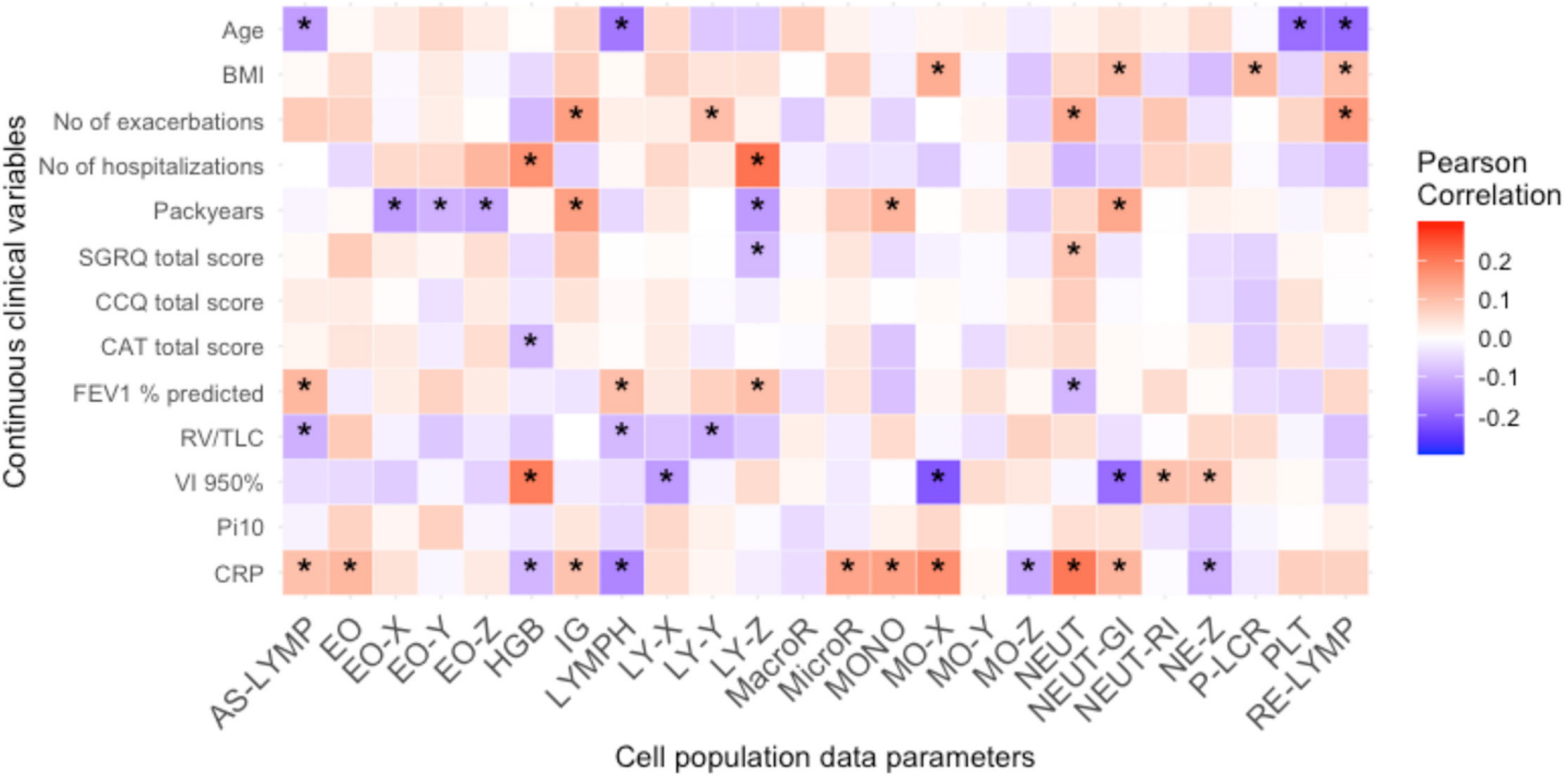
Heatmap showing Pearson correlation coefficients between continuous clinical variables (Y‐axis) and cell population data (X‐axis). Red shades indicate positive correlations, blue shades indicate negative correlations, and white reflects no correlations. The strength of the correlation is indicated by the colour intensity. * = *p* < 0.05.

Pearson's correlation between continuous cell population parameters and continuous clinical variables is presented in Figure 3 and Table S4. Several significant but weak associations were identified. Higher neutrophil counts (NEUT) were negatively correlated with FEV_1_%predicted (r = −0.10, *p* = 0.034), and immature granulocytes (IG) and reactive lymphocytes (RE‐LYMP) showed weak positive correlations with the number of exacerbations in the previous year (r = 0.15, *p* = 0.001 and r = 0.16, *p* < 0.001, respectively).

### 3.3 Cluster Analysis of the Cell Population Parameters

**FIGURE 4:**
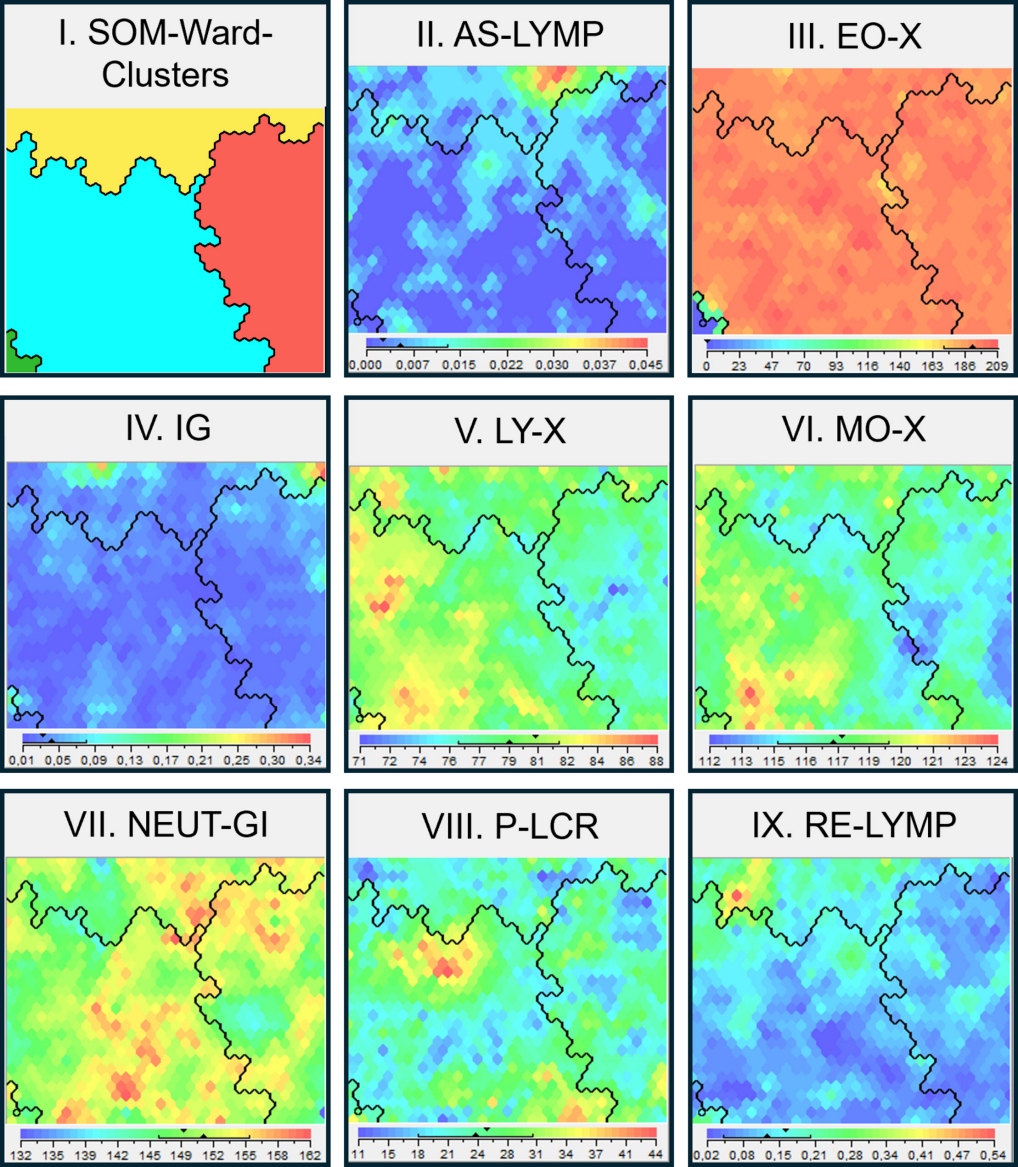
Visual representation of the four clusters derived from cell population data (CPD). (I) Four clusters were identified, the *normal cell counts, resting pattern* cluster in red (*n* = 156), the *normal cell counts, activated pattern* cluster depicted in blue (*n* = 241), and the *elevated cell counts, activated pattern* cluster depicted in yellow (*n* = 97), and the *low‐eosinophilic* cluster depicted in green (*n* = 5). The method mapped all patients according to their cell population characteristics, placing similar subjects in proximity, and those with greater differences are positioned further apart. The colours in the legend of each separate map (II–IX) represent the size of the attribute, with blue indicating low values, and red high values. (II) AS‐LYMP, antibody‐synthesising lymphocytes (10^9^/L); (III) EO‐X, side scattered light distribution of the eosinophil population (channel); (IV) IG, immature granulocytes (10^9^/L); (V) LY‐X, side scattered light distribution of the lymphocyte population (channel); (VI) MOX‐X, side scattered light distribution of the monocyte population (channel); (VII) NEUT‐GI, neutrophil granularity intensity (scatter intensity); (VIII) P‐LCR, platelet large cell ratio (%); (IX) RE‐LYMP, reactive lymphocytes (10^9^/L).

A cluster analysis based on the selected CPD parameters identified four distinct clusters (Figure 4, Figure S1).

The *normal cell counts, resting pattern* cluster (*n* = 156) was defined by generally normal counts and low activity of inflammatory cells. This cluster exhibited normal values for antibody‐synthesising lymphocytes (AS‐LYMP) and reactive lymphocytes (RE‐LYMP), lower granularity of the lymphocytes (LY‐X), monocytes (MO‐X), and neutrophils (NEUT‐GI), lower nucleic acid content levels of lymphocytes (LY‐Y), monocytes (MO‐Y), and neutrophils (NEUT‐RI), and smaller sizes of the lymphocytes (LY‐Z), monocytes (MO‐Z), and neutrophils (NE‐Z). Macrocytic red blood cells (MacroR) numbers were also lower, while haemoglobin concentration (HGB), absolute lymphocytes (LYMPH), and platelet (PLT) counts were elevated. Summarising, this cluster represents a low systemic inflammatory group of COPD patients.

The *normal cell counts, activated pattern* (*n* = 241) were characterised by highly activated inflammatory cells, such as higher granularity of the eosinophils (EO‐X), lymphocytes (LY‐X), monocytes (MO‐X), and neutrophils (NEUT‐GI), increased nucleic acid content of eosinophils (EO‐Y), lymphocytes (LY‐Y), and neutrophils (NEUT‐RI), as well as increased size of the eosinophils (EO‐Z), lymphocytes (LY‐Z), and neutrophils (NE‐Z). Meanwhile, the absolute counts of inflammatory cells, such as lymphocytes (LYMPH), monocytes (MONO), and neutrophils (NEUT), were normal. Additionally, platelet count (PLT) and microcytic red blood cell counts (MicroR) were normal in this cluster. Summarising, this represents a group of subjects with normal systemic numbers but highly active inflammatory cells.

The *elevated cell counts, activated pattern* cluster (*n* = 97) was represented by higher absolute counts of inflammatory cells, such as antibody‐synthesising lymphocytes (AS‐LYMP), eosinophils (EO), immature granulocytes (IG), lymphocytes (LYMPH), monocytes (MONO), neutrophils (NEUT), and reactive lymphocytes (RE‐LYMP). Additionally, it was characterised by higher activity values of inflammatory cells, reflected by high granularity of the lymphocytes (LY‐X) and monocytes (MO‐X), and increased nucleic acid content of lymphocytes (LY‐Y) and monocytes (MO‐Y). Platelet count (PLT) and microcytic red blood cell counts (MicroR) were elevated as well in this cluster, whereas haemoglobin concentration (HGB), size of the neutrophils, and platelet to large cell ratio (P‐LCR) were low. Summarising, this cluster represents a high systemic inflammatory group of COPD patients.

The *low‐eosinophilic* cluster (*n* = 5), the smallest cluster, was characterised by low absolute counts of monocytes (MONO) and eosinophils (EO), along with low eosinophilic granularity (EO‐X), decreased eosinophil nucleic acid content (EO‐Y), and decreased eosinophil size (EO‐Z).

### 3.4 Differences in Clinical Characteristics Using Cell Population Data (CPD) Derived Clusters

Due to the small number of patients, *the low‐eosinophilic* cluster was excluded from the analysis; therefore, three major clusters were compared in terms of clinical parameters (Table 2).

**TABLE 2:** Clinical characteristics of the severe COPD patients, stratified for the cell population‐derived clusters.

	Normal cell counts, resting pattern	Normal cell counts, activated pattern	Elevated cell counts, activated pattern	*p*
*n*	156	241	97	
Age, years	61.6 ± 7.9	62.5 ± 7.0 ^3^	59.9 ± 7.3 ^2^	**0.012**
Sex, female	105 (67)	162 (67)	75 (77)	0.157
BMI, kg/m^2^	24.1 ± 4.03	24.2 ± 3.98	24.9 ± 4.81	0.242
Smoking status				0.626
Current smoker	3 (2)	5 (2)	1 (1)	
Ex‐smoker	151 (97)	236 (98)	95 (99)	
Never smoker	1 (1)	0 (0)	0 (0)	
Packyears	39.8 ± 18.5	39.8 ± 18.5	43.6 ± 20.4	0.210
Patients using ICS or ICS/LABA	132 (96)	198 (93)	87 (99)	0.068
Number of exacerbations in the previous year	2.00 [1.00, 3.00]	1.00 [1.00, 3.00] ^3^	2.00 [1.00, 3.00] ^2^	**0.020**
Hospitalisation due to an exacerbation in the previous year				0.363
Yes	54 (73)	90 (74)	44 (86)	
No	18 (24)	28 (23)	7 (14)	
Unknown	2 (3)	4 (3)	0 (0.0)	
Number of hospitalizations due to an exacerbation in the previous year	0.45 ± 1.2	0.46 ± 1.0	0.51 ± 0.84	0.906
CAT total score	22.3 ± 5.7	21.6 ± 6.1	21.8 ± 6.2	0.591
CCQ total score	3.08 ± 0.89	2.96 ± 0.91	3.08 ± 0.88	0.394
mMRC	3.00 [2.00, 3.00]	3.00 [2.00, 3.00]	2.00 [2.00, 3.00]	0.142
SGRQ total score	59.1 ± 12.6	56.2 ± 13.3	57.3 ± 13.0	0.105
FEV_1_% predicted	27.6 ± 9.9	29.8 ± 10.1	29.5 ± 8.3	0.073
RV/TLC, %	62.3 ± 8.7 ^3^	60.3 ± 8.8	59.8 ± 7.6 ^1^	**0.038**
Emphysema destruction severity, LAA < −950 HU	36.3 ± 7.9 ^3^	35.0 ± 8.9	32.79 ± 9.3 ^1^	**0.011**
Pi10	2.64 ± 0.31	2.62 ± 0.30	2.66 ± 0.29	0.608
Chronic kidney disease	7 (5)	6 (3)	3 (3)	0.561
Congestive heart failure	4 (3)	5 (2)	2 (2)	0.943
Pulmonary arterial hypertension	11 (8)	17 (8)	4 (5)	0.516
Atherosclerosis	32 (23)	36 (17)	21 (24)	0.285
Myocardial infarction	14 (10) ^2^	4 (2) ^1,3^	8 (9) ^2^	**0.003**
Coronary artery disease	20 (15)	15 (7)	12 (14)	0.065
Cerebrovasculair accident	8 (6)	4 (2)	2 (2)	0.114
Malignancy	19 (14)	42 (20)	14 (16)	0.282
Autoimmune disorder	10 (7)	12 (6)	6 (7)	0.849
Hypertension	51 (37)	68 (33)	28 (32)	0.624
Diabetes mellitus	5 (4)	9 (4)	8 (9)	0.154
CRP, mg/L	3.66 ± 4.53 ^2^	2.79 ± 2.81 ^1,3^	4.16 ± 3.81 ^2^	**0.003**

*Note*: Data are presented as *n*, *n* (%), mean ± SD or median [interquartile range], unless otherwise specified. Differences were tested with ANOVA or Kruskal–Wallis, post‐hoc pairwise comparisons were performed with Bonferroni correction. Categorical variables were tested using Chi‐square tests. ^123^: statistically significant differences between clusters are indicated with the corresponding superscript. Significant *P* values lower than 0.05 are prestented in bold.

Abbreviations: BMI, Body Mass Index; CAT, COPD assessment test; CCQ, Clinical COPD Questionnaire; CRP, C‐reactive protein; FEV_1_, forced expiratory volume in 1 s; ICS, inhaled corticosteroids; LAA, low attenuation areas < −950 hounsfield units (HU) on the inspiratory CT scan; LABA, long acting β2 agonist; mMRC, Modified Medical Research Council Dyspnoea Scale; Pi10, 10‐mm internal luminal perimeter; RV/TLC, residual volume/total lung capacity; SGRQ, St. George's Respiratory Questionnaire.

Patients in the *normal cell counts, resting pattern* cluster (67% female, mean ± SD age 61.6 ± 7.9 years, 1.9% current smokers, FEV_1_ 27.6% ± 9.9% predicted) exhibited the highest degree of emphysema severity. Additionally, RV/TLC was increased, indicating more hyperinflation compared to the other clusters. Notably, patients in the *normal counts, resting pattern* cluster had a significantly higher prevalence of myocardial infarction (10.2%) and, however, not significant, more coronary artery disease (14.6%) compared to patients in the *normal cell counts, activated pattern* cluster.

The *normal cell counts, activated pattern* cluster (67% female, mean ± SD age 62.5 ± 7.0 years, 2.1% current smokers, FEV_1_ 29.8% ± 10.1% predicted) was characterised by the highest age, the lowest number of COPD exacerbations in the past year, the fewest occurrence of myocardial infarctions, and the lowest CRP‐levels.

The *elevated cell counts, activated pattern* cluster (77% female, mean ± SD age 59.9 ± 7.3 years, 1.0% current smokers, FEV_1_ 29.5% ± 8.3% predicted) comprised the youngest patients with the highest number of exacerbations and hospitalizations due to exacerbations. Compared to the *normal cell counts, resting pattern* cluster and the *normal cell counts, activated pattern* cluster, the *elevated cell counts, activated pattern* cluster had the lowest degree of emphysema severity and the highest CRP levels. Additionally, myocardial infarction was significantly more common in the *elevated cell counts, activated pattern* cluster (9%) compared to *normal cell counts, activated pattern* cluster (2%).

## 4 Discussion

This study demonstrated that although most individual cell population parameters showed limited deviation from established reference ranges, the combination of both cell counts and activation markers revealed clinically meaningful patterns. Three distinct inflammatory subgroups, derived from CPD, were identified among patients with severe COPD. These included a *normal cell counts, resting pattern* cluster, a *normal cell counts, activated pattern* cluster, and an *elevated cell counts, activated pattern* cluster. Each subgroup was associated with a distinct clinical profile. Together, the findings of this study suggest that composite inflammatory profiles, including both quantitative and qualitative cellular characteristics, can offer deeper insights into disease mechanisms and patient subtypes.

The cell population parameters showed various associations with clinical characteristics. Multiple parameters correlated with inflammatory‐related characteristics, such as the CRP levels, age, or the number of pack‐years. Interestingly, it was found that the severity of emphysema was positively correlated with haemoglobin levels and negatively correlated with the side scatter of monocytes, lymphocytes, and neutrophils. On the other hand, lung function, as determined by the FEV_1_, was positively correlated with the number of lymphocytes, antibody synthesising lymphocytes, and the size of lymphocytes, and negatively correlated with the number of circulating neutrophils. These results indicate that different clinical phenotypes of COPD are related to different underlying inflammatory profiles. However, it has to be considered that these correlations were identified in a homogenous cohort consisting only of patients with severe COPD and that the obtained Pearson's coefficients were rather low (±0.1–0.2), signalling statistically significant but weak correlations. To gain more insight into the relation between cell population parameters and clinical phenotypes, a cluster analysis was performed based on the cell population parameters.

Surprisingly, the *normal cell counts, resting pattern cluster*, exhibited more advanced structural lung disease and symptoms, such as emphysema, hyperinflation, and dyspnoea, despite low systemic inflammation (both in cell counts and activation markers). This may suggest that systemic inflammation may not be the primary driver of disease pathology in this group. Instead, high CT emphysema scores and increased RV/TLC may indicate a lung tissue destruction phenotype rather than an inflammation‐driven subtype. Consequently, if this hypothesis holds true, systemic anti‐inflammatory treatments may be less effective, whereas therapies targeting lung mechanics, such as bronchodilators, lung volume reduction, and rehabilitation, may be more beneficial in this subtype. Notably, this cluster also had a relatively high prevalence of myocardial infarction and coronary artery disease, similar to the *elevated counts, activated pattern* cluster. While systemic inflammation is a well‐recognised risk factor for cardiovascular disease in COPD [22, 23]. other mechanisms, such as hyperinflation increasing left ventricular mass and coronary calcification [24], may play a role in this group.

Patients in the *normal cell counts, activated pattern* cluster, appeared clinically more stable than those in other clusters, with less airway obstruction, fewer exacerbations, and the lowest prevalence of myocardial infarctions. It may be that inflammation in these patients is well‐regulated. Activated eosinophils, lymphocytes, and neutrophils could reflect an effective immune response that effectively clears infections and reduces exacerbations. Prior studies have linked eosinophilic activation in COPD to improved pathogen clearance and steroid responsiveness [25, 26]. Alternatively, the absolute number of inflammatory cells may be too low to cause significant pathology, despite increased activation. Notably, our analysis was limited to peripheral blood, and the immune activity in lung tissue may differ.

Patients with *elevated cell counts, activated pattern* cluster, experienced more frequent exacerbations, possibly associated with the increased number and activity of neutrophils, monocytes, eosinophils, and reactive lymphocytes, cells known to be involved in triggering COPD exacerbations [27, 28]. These patients also more often had a history of myocardial infarction, suggesting a link between systemic inflammation and cardiovascular disease. Chronic inflammation promotes cardiovascular disease with monocytes driving plaque formation [29, 30]. While neutrophils contribute to early vascular inflammation and plaque instability [31, 32]. Elevated platelet counts and reduced P‐LCR in this cluster may have further reflected a pro‐thrombotic state, contributing to cardiovascular risk [33]. Interestingly, despite their inflammatory burden, patients in this cluster exhibited less emphysema and less airflow obstruction compared to patients in the *normal cell counts, resting pattern* cluster, suggesting that this phenotype may be primarily inflammation‐driven rather than driven by structural lung destruction. The last cluster that was identified was the *low‐eosinophilic cluster*, a small cluster containing only 5 severe COPD subjects. Nevertheless, this cluster has distinct characteristics, as the number and activity of circulating eosinophils were lower compared to the other clusters. Due to the low number of subjects in this cluster, it was not possible to assess the clinical relevance of this cluster. Future studies, using a larger cohort with more patients with inactive circulating eosinophils, are needed to evaluate the relevance of this cluster.

In order to further evaluate the clinical relevance of the identified clusters and to understand the underlying pathways, future studies are needed. These studies should focus on identifying transcriptional and proteomic differences in patients from the different clusters to identify genes and pathways related to each cluster. Additionally, functional studies are needed to fully understand the role of specific CPD parameters in relation to the associated clinical characteristics.

A key strength of this study is the large cohort of extensively characterised severe COPD patients, in whom CPD were collected. This enabled the identification of distinct inflammatory subgroups beyond conventional cell counts, which have not been previously investigated in COPD. However, the cross‐sectional design of this study prevents conclusions about the stability of the clusters over time, the longitudinal changes in cell population parameters, and their predictive value over time. Furthermore, our data were not validated using an independent cohort. Another limitation of this study was the exclusion of COPD patients using oral prednisone. This decision was made as systemic corticosteroids can suppress inflammatory markers; however, exclusion may have led to the underrepresentation of patients with an eosinophilic subtype, as these patients are more likely to be treated with oral corticosteroids. Moreover, as our analysis was limited to severe COPD patients, future research should explore cell population parameters in mild and moderate COPD or at‐risk control populations to assess whether certain immune profiles predispose individuals to developing or progressing COPD and therefore serve as possible prognostic markers.

In conclusion, this is the first study to demonstrate that cell population parameters can be used to identify clinically relevant clusters in severe COPD patients. These clusters differ in immune activation and clinical characteristics. As an easily accessible and cost‐effective biomarker, CPD has the potential to provide additional insights beyond inflammatory cell counts and improve patient classification and clinical decision‐making.

## Author Contributions

**Pauline J. M. Kuks:** investigation, writing – original draft, visualization, formal analysis, data curation. **Jorine E. Hartman:** formal analysis, writing – review and editing. **Else A. M. D. ter Haar:** formal analysis, writing – review and editing. **L. Joost van Pelt:** resources, data curation, methodology, writing – review and editing. **Dirk‐Jan Slebos:** supervision, resources, writing – review and editing. **Maarten van den Berge:** resources, supervision, writing – review and editing. **Simon D. Pouwels:** conceptualization, investigation, methodology, validation, writing – review and editing, formal analysis, data curation, supervision.

## Ethics Statement

The GSCC cohort was approved by the ethics committee of the University Medical Center Groningen (EC number: 2014/102), and all participants provided written informed consent. The study was registered at clinicaltrials.gov [NCT04023409].

## Conflicts of Interest

Maarten van den Berge is an Editorial Board member of Respirology and a co‐author of this article. He was excluded from all editorial decision‐making related to the acceptance of this article for publication. The other authors declare not to have any conflicts of interest that may be considered to influence directly or indirectly the content of the manuscript.

## Supporting information


**Figure S1:** Graphic presentation of the clusters.
**Table S1:** Patient baseline demographics.
**Table S2:** Summary associations between cell population data parameters and clinical characteristics.
**Table S3:1–26:** Univariate results of the association between cell population parameters and clinical characteristics.
**Table S4:** (a,b) Pearson correlation coefficients and *p*‐values between continuous clinical variables and cell population data parameters.

## Data Availability

The data that support the findings of this study are available on request from the corresponding author. The data are not publicly available due to privacy or ethical restrictions.
