# Supplementary material for: Identification of Clinically Distinct Clusters in Patients With Severe COPD Using Circulating Blood Cell Population Parameters
Source: Respirology. 2025 Oct 19;31(2):141–51. doi: 10.1002/resp.70146 (PMC12865525; doi:10.1002/resp.70146)
Supplement: Supplementary file 1 — Figure S1: Graphic presentation of the clusters. Table S1: Patient baseline demographics. Table S2: Summary associations between cell population data parameters and clinical characteristics. Table S3:1–26: Univariate results of the association between cell population parameters and clinical characteristics. Table S4: (a,b) Pearson correlation coefficients and p‐values between continuous clinical variables and cell population data parameters. [file RESP-31-141-s001.docx]

**Identification of Clinically Distinct Clusters in Patients with Severe COPD Using Circulating Blood Cell Population Parameters**

Pauline J.M. Kuks^1,2^, Jorine E. Hartman^1,2^, Else A.M.D. ter Haar^1,2^, L. Joost van Pelt^3^, Dirk-Jan Slebos^1,2^, Maarten van den Berge^1,2^, Simon D. Pouwels^1,2,4^

^1^ University of Groningen, University Medical Center Groningen, Department of Pulmonary Diseases, Groningen, The Netherlands

^2^ University of Groningen, University Medical Center Groningen, Groningen Research Institute for Asthma and COPD, Groningen, The Netherlands

^3^ University of Groningen, University Medical Center Groningen, Department of Laboratory Medicine, Groningen, The Netherlands

^4^ University of Groningen, University Medical Center Groningen, Department of Pathology and Medical Biology, Groningen, The Netherlands

**Supplementary data**

**Table of contents**

[Supplementary Table S1: Patient baseline demographics 3](#_Toc210211728)

[Supplementary Table S2: summary supplementary table 3.1-3.26 - associations between cell population data parameters and clinical characteristics 4](#_Toc210211729)

[Supplementary Table S3.1: Baseline characteristics by Antibody-synthesizing lymphocytes (AS-LYMP) 7](#_Toc210211730)

[Supplementary Table S3.2: Baseline characteristics by Eosinophils (absolute count) (EO) 9](#_Toc210211731)

[Supplementary Table S3.3: Baseline characteristics by Side scattered light distribution width of de eosinophil population (EO-X) 11](#_Toc210211732)

[Supplementary Table S3.4: Baseline characteristics by Fluorescent light distribution width of the eosinophil population (EO-Y) 13](#_Toc210211733)

[Supplementary Table S3.5: Baseline characteristics by Forward scattered light distribution width of the eosinophil population (EO-Z) 16](#_Toc210211734)

[Supplementary Table S3.6: Baseline characteristics by Hemoglobin concentration as measured in the RBC/PLT channel (HGB) 18](#_Toc210211735)

[Supplementary Table S3.7: Baseline characteristics by Immature granulocytes (IG) 20](#_Toc210211736)

[Supplementary Table S3.8: Baseline characteristics by Lymphocytes (absolute count) (LYMPH) 22](#_Toc210211737)

[Supplementary Table S3.9: Baseline characteristics by Lymphocytes (absolute count) 24](#_Toc210211738)

[Supplementary Table S3.10: Baseline characteristics by Side scattered light distribution width of de lymphocyte population (LY-X) 26](#_Toc210211739)

[Supplementary Table S3.11: Baseline characteristics by Side scattered light distribution width of de lymphocyte population (LY-X) 28](#_Toc210211740)

[Supplementary Table S3.12: Baseline characteristics by Fluorescent light distribution width of the lymphocyte population (LY-Y) 30](#_Toc210211741)

[Supplementary Table S3.13: Baseline characteristics by Forward scattered light distribution width of the lymphocyte population (LY-Z) 32](#_Toc210211742)

[Supplementary Table S3.14: Baseline characteristics by Macrocytic red blood cells (MacroR) 34](#_Toc210211743)

[Supplementary Table S3.15: Baseline characteristics by Microcytic red blood cells (MicroR) 36](#_Toc210211744)

[Supplementary Table S3.16: Baseline characteristics by Monocytes (absolute count) (MONO) 38](#_Toc210211745)

[Supplementary Table S3.17: Baseline characteristics by Side scattered light distribution width of de monocyte population (MO-X) 40](#_Toc210211746)

[Supplementary Table S3.18: Baseline characteristics by Side scattered light distribution width of de monocyte population (MO-X) 42](#_Toc210211747)

[Supplemenentary Table S3.19: Baseline characteristics by Fluorescent light distribution width of the monocyte population (MO-Y) 44](#_Toc210211748)

[Supplementary Table S3.20: Baseline characteristics by Neutrophils (absolute count) (NEUT) 46](#_Toc210211749)

[Supplementary Table S3.21: Baseline characteristics by Mean forward scattered light intensity of the neutrophil population (NE-FSC) 48](#_Toc210211750)

[Supplementary Table S3.22: Baseline characteristics by Neutrophil reactivity intensity (Neutrophil reactivity intensity) 50](#_Toc210211751)

[Supplementary Table S3.23: Baseline characteristics by Neutrophil granularity intensity (NEUT-GI) 52](#_Toc210211752)

[Supplementary Table S3.24: Baseline characteristics by Platelet large cell ratio (P-LCR) 54](#_Toc210211753)

[Supplementary Table S3.25: Baseline characteristics by Platelet count (PLT) 56](#_Toc210211754)

[Supplementary Table S3.26: Baseline characteristics by Reactive lymphocytes (RE-LYMP) 58](#_Toc210211755)

[Supplementary Table S4a: Pearson correlation coefficients and p-values between continuous clinical variables and cell population data parameters 60](#_Toc210211756)

[Supplementary Table S4b: Pearson correlation coefficients and p-values between continuous clinical variables and cell population data parameters 61](#_Toc210211757)

[Supplementary Figure S1: Graphic presentation of the clusters. 65](#_Toc210211758)

# Supplementary Table S1: Patient baseline demographics

| Patients | 496 |
| --- | --- |
| Age, years | 61.7±7.43 |
| Sex, female | 344 (69) |
| BMI, kg/m^2^ | 24.3±4.26 |
| Smoking status |  |
| Current smoker | 9 (2) |
| Ex-smoker | 484 (98) |
| Never smoker | 1 (0) |
| Packyears | 40.8±19.5 |
| Patients Using ICS or ICS/LABA | 418 (95) |
| Number of exacerbations in the previous year | 2.00 [1.00, 3.00] |
| SGRQ total score | 57.1±13.2 |
| FEV_1_ % predicted | 29.0±9.79 |
| RV/TLC, % | 60.9±8.62 |
| Data are presented as n, n (%), mean±SD or median [interquartile range], unless otherwise specified. BMI: Body Mass Index; ICS/LABA: inhaled corticosteroids/long acting beta2 agonist; FEV_1_:Forced expiratory volume in 1 second; SGRQ: St George’s Respiratory Questionnaire; RV/TLC: Residual volume/total lung capacity | |

| Supplementary Table S2: summary supplementary table 3.1-3.26 - associations between cell population data parameters and clinical characteristics | | | | | |
| --- | --- | --- | --- | --- | --- |
|  | **Below, n (%)** | **Normal, n (%)** | **Above, n (%)** | **Summary clinical findings *** | |
| AS-LYMP (Supplementary Table 3.1) | 0 | 273 (56%) | 213 (44%) | ↓ Age  ↑ FEV1 %predicted  ↓ RV/TLC (%) | |
| EO (Supplementary Table 3.2) | 7 (1) | 424 (86) | 64 (13) | ↑ Age  ↑Myocardial infarction | |
| EO-X (Supplementary Table 3.3) | 28 (5) | 450 (91) | 18 (4) | - | |
| EO-Y (Supplementary Table 3.4) | 15 (3) | 412 (83) | 69 (14) | - | |
| EO-Z (Supplementary Table 3.5) | 15 (3) | 392 (79) | 89 (18) | ↓ Atherosclerosis | |
| HGB  (Supplementary Table 3.6) | 17 (3) | 428 (86) | 53 (11) | - | |
| IG  (Supplementary Table 3.7) | 0 (0) | 446 (90) | 49 (10) | ↑Number of exacerbations in the previous year | |
| LYMPH  (Supplementary Table 3.8 + 3.9) | 39 (8) | 426 (86) | 30 (6) | *Comparing* ***below*** *to normal* | *Comparing* ***above*** *to normal* |
|  |  |  |  | ↑ Age  ↓ Female  ↑Number of hospitalization due to an exacerbation in the previous year | ↑ number of exacerbations in the previous year |
| LY-X  (Supplementary Table 3.10 + 3.11) | 25 (5) | 328 (66) | 142 (29) | *Comparing* ***below*** *to normal* | *Comparing* ***above*** *to normal* |
|  |  |  |  | ↑*Current smokers* | ↓ Female  ↓ ICS or ICS-LABA use |
| LY-Y (Supplementary Table 3.12) | 1 (0) | 324 (66) | 170 (34) | ↑ICS or ICS-LABA use | |
| LY-Z  (Supplementary Table 3.13) | 317 (64) | 170 (34) | 8 (2) | ↑Packyears  ↑ Hospitalization due to an exacerbation in the previous year  ↓FEV1 %predicted  ↓ Diabetes Mellitus | |
| MacroR  (Supplementary Table 3.14) | 1 (0) | 431 (87) | 66 (13) | ↓ Female  ↑ *Current smokers*  ↑ Myocardial infarction | |
| MicroR  (Supplementary Table 3.15) | 3 (0) | 462 (93) | 33 (7) | ↑CAT | |
| MONO  (Supplementary Table 3.16) | 12 (2) | 375 (76) | 108 (22) | ↓ Current smokers  ↑ CRP | |
| MO-X  (Supplementary Table 3.17 + 3.18) | 86 (17) | 387 (78) | 22 (5) | Above:  ↓ ICS or ICS-LABA use  ↑ Atherosclerosis  Below:  ↓ Emphysema destruction severity, LAA -950HU  ↑ Myocardial infarction  ↑ Coronary Artery Disease | |
| MO-Y  (Supplementary Table 3.19) | 5 (1) | 387 (78) | 103 (21) | ↑ Pulmonary Arterial Hypertension | |
| MO-Z | 12 (2) | 479 (97) | 4 (1) | - | |
| NEUT  (Supplementary Table 3.20) | 0 (0) | 308 (62) | 187 (38) | ↓ FEV1 %predicted  ↑ CRP | |
| NE-Z  (Supplementary Table 3.21) | 96 (19) | 394 (80) | 5 (1) | ↑ CRP | |
| NEUT-RI  (Supplementary Table 3.22) | 19 (4) | 437 (88) | 39 (8) | ↑ Emphysema destruction severity, LAA -950HU | |
| NEUT-GI  (Supplementary Table 3.23) | 15 (3) | 440 (89) | 40 (8) | ↓ICS or ICS-LABA use | |
| P-LCR  (Supplementary Table 3.24) | 111(22) | 386 (78) | 0 (0) | ↓ BMI | |
| PLT  (Supplementary Table 3.25) | 8 (2) | 440 (88) | 50 (10) | ↓ Age | |
| RE-LYMP  (Supplementary Table 3.26) | 9 (2) | 367 (76) | 110 (23) | ↓ Age  ↑ Female  ↑ BMI  ↑ Number of exacerbations in the previous year  ↑ CRP | |
| BMI: Body Mass Index; ICS/LABA: inhaled corticosteroids/long acting beta2 agonist; FEV_1_:Forced expiratory volume in 1 second; RV/TLC: Residual volume/total lung capacity.  * Summary clinical findings reflect differences between the group outside the reference interval (either below or above) and the group within the normal reference interval. Comparisons were only performed if the proportion of patients in the abnormal group was ≥5%. | | | | | |

## Supplementary Table S3.1: Baseline characteristics by Antibody-synthesizing lymphocytes (AS-LYMP)

|  | **Above reference interval** | **Within normal reference interval** | **p** |
| --- | --- | --- | --- |
| n | 213 | 273 |  |
| Age, years | 60.76 ±7.67 | 62.50 60.76) | **0.010** |
| Sex, female | 150 (70.4) | 187 (68.5) | 0.721 |
| BMI, kg/m2 | 24.37 ±4.30 | 24.19 ±4.17 | 0.637 |
| Smoking status |  |  | 0.431 |
| Current smoker | 3 (1.4) | 6 (2.2) |  |
| Ex-smoker | 208 (98.1) | 266 (97.8) |  |
| Never smoker | 1 (0.5) | 0 (0.0) |  |
| Packyears | 40.67 (19.14) | 41.17 (19.84) | 0.780 |
| Use of ICS or ICS/LABA | 178 (96.2) | 231 (93.5) | 0.309 |
| Number of exacerbations in the previous year | 1.00 [1.00, 3.00] | 2.00 [1.00, 3.00] | 0.953 |
| Hospitalization due to an exacerbation in the previous year |  |  | 0.509 |
| Yes | 77 (75.5) | 107 (77.0) |  |
| No | 24 (23.5) | 28 (20.1) |  |
| Unknown | 1 (1.0) | 4 (2.9) |  |
| Number of hospitalizations in the previous year | 0.37 ±0.75 | 0.55 ±1.23 | 0.068 |
| CAT total score | 21.82 ±6.31 | 21.80 ±5.69 | 0.963 |
| CCQ total score | 3.04 ±0.96 | 3.00 ±0.84 | 0.680 |
| mMRC | 3.00 [2.00, 3.00] | 3.00 [2.00, 3.00] | 0.767 |
| SGRQ total score | 57.03 ±13.11 | 57.28 ±13.17 | 0.841 |
| FEV1 % predicted | 30.17 ±9.66 | 28.13 ±9.79 | **0.022** |
| RV/TLC, % | 59.91 ±8.48 | 61.52 ±8.68 | **0.041** |
| Emphysema destruction severity, LAA -950HU | 34.74 ±8.87 | 35.41 ±8.77 | 0.419 |
| Pi10 | 2.63 ±0.29 | 2.64 ±0.31 | 0.920 |
| Chronic kidney disease | 5 (2.7) | 11 (4.6) | 0.456 |
| Congestive heart failure | 5 (2.7) | 6 (2.5) | 1.000 |
| Pulmonary Arterial Hypertension | 9 (4.9) | 22 (9.1) | 0.136 |
| Atherosclerosis | 40 (21.6) | 47 (19.5) | 0.677 |
| Myocardial Infarction | 14 (7.6) | 12 (5.0) | 0.367 |
| Coronary Artery disease | 21 (11.4) | 26 (10.8) | 0.978 |
| Cerebrovasculair accident | 7 (3.8) | 6 (2.5) | 0.627 |
| Malignancy | 26 (14.1) | 48 (19.9) | 0.146 |
| Autoimmune disorder | 13 (7.0) | 15 (6.2) | 0.893 |
| Hypertension | 63 (34.1) | 82 (34.0) | 1.000 |
| Diabetes Mellitus | 11 (5.9) | 10 (4.1) | 0.533 |
| CRP, mg/L | 3.67 ±3.92 | 3.05 ±3.34 | 0.064 |
| Data are presented as n, n (%), mean±SD or median [interquartile range], unless otherwise specified. Data are presented as n, n (%), mean±SD or median [interquartile range], unless otherwise specified. Univariate differences were tested with Chi-square tests, independent T-tests and Mann-whitney U-tests as appropriate. BMI: Body Mass Index; ICS: inhaled corticosteroids; LABA: long acting β2 agonist; CAT: COPD assessment test; CCQ: Clinical COPD questionnaire; CCQ: Clinical COPD Questionnaire; mMRC: Modified Medical Research Council Dyspnea Scale; SGRQ: St. George’s Respiratory Questionnaire; FEV_1_: Forced Expiratory Volume in 1 second; RV/TLC: Residual Volume/Total Lung Capacity; LAA: low attenuation areas < −950 hounsfield units (HU) on the inspiratory CT scan; Pi10: 10-mm internal luminal perimeter; CRP: C-Reactive Protein. | | | |

## Supplementary Table S3.2: Baseline characteristics by Eosinophils (absolute count) (EO)

|  | **Below reference interval** | **Within normal reference interval** | **p** |
| --- | --- | --- | --- |
| n | 64 | 424 |  |
| Age, years | 63.34 ±6.82 | 61.38 ±7.48 | **0.048** |
| Sex, female | 39 (60.9) | 301 (71.0) | 0.138 |
| BMI, kg/m2 | 23.42 ±4.08 | 24.41 ±4.25 | 0.082 |
| Smoking status |  |  | 0.911 |
| Current smoker | 1 (1.6) | 8 (1.9) |  |
| Ex-smoker | 63 (98.4) | 413 (97.9) |  |
| Never smoker | 0 (0.0) | 1 (0.2) |  |
| Packyears | 42.95 ±23.30 | 40.49 ±18.91 | 0.350 |
| Use of ICS or ICS/LABA | 48 (94.1) | 362 (94.8) | 1.000 |
| Number of exacerbations in the previous year | 2.00 [1.00, 3.00] | 2.00 [1.00, 3.00] | 0.906 |
| Hospitalization due to an exacerbation in the previous year |  |  | 0.679 |
| Yes | 25 (78.1) | 161 (76.3) |  |
| No | 7 (21.9) | 45 (21.3) |  |
| Unknown | 0 (0.0) | 5 (2.4) |  |
| Number of hospitalizations in the previous year | 0.47 ±0.86 | 0.47 ±1.08 | 0.998 |
| CAT total score | 22.75 ±6.17 | 21.67 ±5.97 | 0.195 |
| CCQ total score | 3.05 ±1.00 | 3.02 ±0.88 | 0.810 |
| mMRC | 3.00 [2.00, 3.00] | 3.00 [2.00, 3.00] | 0.496 |
| SGRQ total score | 57.73 ±14.69 | 57.18 ±12.94 | 0.766 |
| FEV1 % predicted | 28.46 ±8.26 | 29.12 ±9.95 | 0.614 |
| RV/TLC, % | 61.50 ±8.67 | 60.63 ±8.62 | 0.449 |
| Emphysema destruction severity, LAA -950HU | 36.30 ±8.60 | 34.95 ±8.78 | 0.265 |
| Pi10 | 2.59 ±0.28 | 2.64 ±0.30 | 0.277 |
| Chronic kidney disease | 0 (0.0) | 16 (4.3) | 0.220 |
| Congestive heart failure | 1 (1.8) | 10 (2.7) | 1.000 |
| Pulmonary Arterial Hypertension | 3 (5.3) | 29 (7.8) | 0.677 |
| Atherosclerosis | 14 (24.6) | 74 (20.0) | 0.537 |
| Myocardial Infarction | 8 (14.0) | 18 (4.9) | **0.017** |
| Coronary Artery disease | 10 (17.5) | 37 (10.0) | 0.142 |
| Cerebrovasculair accident | 1 (1.8) | 13 (3.5) | 0.768 |
| Malignancy | 11 (19.3) | 63 (17.0) | 0.815 |
| Autoimmune disorder | 1 (1.8) | 27 (7.3) | 0.198 |
| Hypertension | 20 (35.1) | 123 (33.2) | 0.901 |
| Diabetes Mellitus | 0 (0.0) | 21 (5.7) | 0.130 |
| CRP, mg/L | 2.67 ±2.66 | 3.39 ±3.73 | 0.135 |
| Data are presented as n, n (%), mean±SD or median [interquartile range], unless otherwise specified. Univariate differences were tested with Chi-square tests, independent T-tests and Mann-whitney U-tests as appropriate. BMI: Body Mass Index; ICS: inhaled corticosteroids; LABA: long acting β2 agonist; CAT: COPD assessment test; CCQ: Clinical COPD questionnaire; CCQ: Clinical COPD Questionnaire; mMRC: Modified Medical Research Council Dyspnea Scale; SGRQ: St. George’s Respiratory Questionnaire; FEV_1_: Forced Expiratory Volume in 1 second; RV/TLC: Residual Volume/Total Lung Capacity; LAA: low attenuation areas < −950 hounsfield units (HU) on the inspiratory CT scan; Pi10: 10-mm internal luminal perimeter; CRP: C-Reactive Protein. | | | |

## Supplementary Table S3.3: Baseline characteristics by Side scattered light distribution width of de eosinophil population (EO-X)

|  | **Below reference interval** | **Within normal reference interval** | **p** |
| --- | --- | --- | --- |
| n | 28 | 450 |  |
| Age, years | 61.11 ±6.89 | 61.72 ±7.42 | 0.671 |
| Sex, female | 21 (75.0) | 314 (69.8) | 0.709 |
| BMI, kg/m2 | 24.39 ±5.76 | 24.27 ±4.15 | 0.887 |
| Smoking status |  |  | 0.727 |
| Current smoker | 0 (0.0) | 9 (2.0) |  |
| Ex-smoker | 28 (100.0) | 439 (97.8) |  |
| Never smoker | 0 (0.0) | 1 (0.2) |  |
| Packyears | 46.38 ±27.67 | 40.52 ±19.02 | 0.127 |
| Use of ICS or ICS/LABA | 18 (94.7) | 384 (95.0) | 1.000 |
| Number of exacerbations in the previous year | 2.00 [0.50, 3.50] | 2.00 [1.00, 3.00] | 0.710 |
| Hospitalization due to an exacerbation in the previous year |  |  | 0.789 |
| Yes | 13 (81.2) | 171 (76.0) |  |
| No | 3 (18.8) | 49 (21.8) |  |
| Unknown | 0 (0.0) | 5 (2.2) |  |
| Number of hospitalizations in the previous year | 0.56 ±0.96 | 0.47 ±1.06 | 0.686 |
| CAT total score | 22.42 ±5.67 | 21.69 ±5.95 | 0.540 |
| CCQ total score | 3.04 ±1.00 | 3.01 ±0.89 | 0.840 |
| mMRC | 3.00 [2.00, 3.00] | 3.00 [2.00, 3.00] | 0.182 |
| SGRQ total score | 57.23 ±16.22 | 57.10 ±12.87 | 0.962 |
| FEV1 % predicted | 27.60 ±8.60 | 29.00 ±9.93 | 0.467 |
| RV/TLC, % | 61.86 ±7.64 | 60.92 ±8.67 | 0.577 |
| Emphysema destruction severity, LAA -950HU | 34.81 ±9.65 | 35.11 ±8.76 | 0.865 |
| Pi10 | 2.67 ±0.35 | 2.63 ±0.30 | 0.581 |
| Chronic kidney disease | 0 (0.0) | 16 (4.1) | 0.644 |
| Congestive heart failure | 1 (4.2) | 10 (2.6) | 1.000 |
| Pulmonary Arterial Hypertension | 1 (4.2) | 30 (7.7) | 0.817 |
| Atherosclerosis | 8 (33.3) | 78 (19.9) | 0.187 |
| Myocardial Infarction | 3 (12.5) | 21 (5.4) | 0.314 |
| Coronary Artery disease | 5 (20.8) | 39 (9.9) | 0.180 |
| Cerebrovasculair accident | 0 (0.0) | 14 (3.6) | 0.720 |
| Malignancy | 5 (20.8) | 64 (16.3) | 0.769 |
| Autoimmune disorder | 0 (0.0) | 26 (6.6) | 0.385 |
| Hypertension | 12 (50.0) | 128 (32.7) | 0.128 |
| Diabetes Mellitus | 0 (0.0) | 20 (5.1) | 0.520 |
| CRP, mg/L | 3.12 ±2.63 | 3.33 ±3.73 | 0.766 |
| Data are presented as n, n (%), mean±SD or median [interquartile range], unless otherwise specified. Univariate differences were tested with Chi-square tests, independent T-tests and Mann-whitney U-tests as appropriate. BMI: Body Mass Index; ICS: inhaled corticosteroids; LABA: long acting β2 agonist; CAT: COPD assessment test; CCQ: Clinical COPD questionnaire; CCQ: Clinical COPD Questionnaire; mMRC: Modified Medical Research Council Dyspnea Scale; SGRQ: St. George’s Respiratory Questionnaire; FEV_1_: Forced Expiratory Volume in 1 second; RV/TLC: Residual Volume/Total Lung Capacity; LAA: low attenuation areas < −950 hounsfield units (HU) on the inspiratory CT scan; Pi10: 10-mm internal luminal perimeter; CRP: C-Reactive Protein. | | | |

## Supplementary Table S3.4: Baseline characteristics by Fluorescent light distribution width of the eosinophil population (EO-Y)

|  | **Above reference interval** | **Within normal reference interval** | **p** |
| --- | --- | --- | --- |
| n | 69 | 412 |  |
| Age, years | 61.87 ±7.72 | 61.63 ±7.39 | 0.807 |
| Sex, female | 49 (71.0) | 287 (69.7) | 0.932 |
| BMI, kg/m2 | 24.35 ±3.67 | 24.25 ±4.30 | 0.855 |
| Smoking status |  |  | 0.882 |
| Current smoker | 1 (1.4) | 8 (2.0) |  |
| Ex-smoker | 68 (98.6) | 401 (97.8) |  |
| Never smoker | 0 (0.0) | 1 (0.2) |  |
| Packyears | 40.33 ±15.74 | 40.55 ±19.29 | 0.929 |
| Use of ICS or ICS/LABA | 56 (100.0) | 349 (93.8) | 0.111 |
| Number of exacerbations in the previous year | 1.00 [1.00, 3.00] | 2.00 [1.00, 3.00] | 0.455 |
| Hospitalization due to an exacerbation in the previous year |  |  | 0.183 |
| Yes | 23 (67.6) | 162 (77.9) |  |
| No | 11 (32.4) | 41 (19.7) |  |
| Unknown | 0 (0.0) | 5 (2.4) |  |
| Number of hospitalizations in the previous year | 0.32 ±0.83 | 0.50 ±1.08 | 0.203 |
| CAT total score | 21.22 ±6.22 | 21.91 ±5.92 | 0.386 |
| CCQ total score | 2.88 ±0.88 | 3.04 ±0.89 | 0.180 |
| mMRC | 3.00 [2.00, 3.00] | 3.00 [2.00, 3.00] | 0.400 |
| SGRQ total score | 56.32 ±13.56 | 57.48 ±12.97 | 0.503 |
| FEV1 % predicted | 30.12 ±9.46 | 28.81 ±9.79 | 0.302 |
| RV/TLC, % | 60.10 ±8.62 | 60.95 ±8.62 | 0.447 |
| Emphysema destruction severity, LAA -950HU | 36.48 ±8.51 | 34.83 ±8.84 | 0.164 |
| Pi10 | 2.65 ±0.28 | 2.63 ±0.30 | 0.767 |
| Chronic kidney disease | 3 (5.1) | 13 (3.6) | 0.853 |
| Congestive heart failure | 0 (0.0) | 10 (2.8) | 0.405 |
| Pulmonary Arterial Hypertension | 8 (13.6) | 24 (6.6) | 0.112 |
| Atherosclerosis | 10 (16.9) | 75 (20.8) | 0.615 |
| Myocardial Infarction | 2 (3.4) | 22 (6.1) | 0.598 |
| Coronary Artery disease | 5 (8.5) | 39 (10.8) | 0.755 |
| Cerebrovasculair accident | 2 (3.4) | 12 (3.3) | 1.000 |
| Malignancy | 13 (22.0) | 60 (16.6) | 0.405 |
| Autoimmune disorder | 3 (5.1) | 25 (6.9) | 0.807 |
| Hypertension | 20 (33.9) | 121 (33.5) | 1.000 |
| Diabetes Mellitus | 3 (5.1) | 19 (5.3) | 1.000 |
| CRP, mg/L | 2.62 ±2.64 | 3.47 ±3.83 | 0.080 |
| Data are presented as n, n (%), mean±SD or median [interquartile range], unless otherwise specified. Univariate differences were tested with Chi-square tests, independent T-tests and Mann-whitney U-tests as appropriate. BMI: Body Mass Index; ICS: inhaled corticosteroids; LABA: long acting β2 agonist; CAT: COPD assessment test; CCQ: Clinical COPD questionnaire; CCQ: Clinical COPD Questionnaire; mMRC: Modified Medical Research Council Dyspnea Scale; SGRQ: St. George’s Respiratory Questionnaire; FEV_1_: Forced Expiratory Volume in 1 second; RV/TLC: Residual Volume/Total Lung Capacity; LAA: low attenuation areas < −950 hounsfield units (HU) on the inspiratory CT scan; Pi10: 10-mm internal luminal perimeter; CRP: C-Reactive Protein. | | | |

## Supplementary Table S3.5: Baseline characteristics by Forward scattered light distribution width of the eosinophil population (EO-Z)

|  | **Above reference interval** | **Within normal reference interval** | **p** |
| --- | --- | --- | --- |
| n | 89 | 392 |  |
| Age, years | 62.07 ±8.19 | 61.59 ±7.30 | 0.586 |
| Sex, female | 65 (73.0) | 270 (68.9) | 0.521 |
| BMI, kg/m2 | 23.96 ±3.92 | 24.39 ±4.25 | 0.388 |
| Smoking status |  |  | 0.855 |
| Current smoker | 2 (2.3) | 7 (1.8) |  |
| Ex-smoker | 86 (97.7) | 383 (98.0) |  |
| Never smoker | 0 (0.0) | 1 (0.3) |  |
| Packyears | 40.24 ±16.83 | 40.79 ±19.28 | 0.806 |
| Use of ICS or ICS/LABA | 75 (96.2) | 332 (94.3) | 0.709 |
| Number of exacerbations in the previous year | 1.00 [1.00, 2.00] | 2.00 [1.00, 3.00] | 0.398 |
| Hospitalization due to an exacerbation in the previous year |  |  | 0.085 |
| Yes | 27 (65.9) | 155 (77.9) |  |
| No | 14 (34.1) | 39 (19.6) |  |
| Unknown | 0 (0.0) | 5 (2.5) |  |
| Number of hospitalizations in the previous year | 0.31 ±0.65 | 0.50 ±1.11 | 0.114 |
| CAT total score | 22.18 ±6.16 | 21.80 ±5.94 | 0.597 |
| CCQ total score | 3.00 ±0.91 | 3.03 ±0.89 | 0.784 |
| mMRC | 3.00 [2.00, 3.00] | 3.00 [2.00, 3.00] | 0.377 |
| SGRQ total score | 56.28 ±13.08 | 57.71 ±12.89 | 0.356 |
| FEV1 % predicted | 29.97 ±9.16 | 28.77 ±9.82 | 0.294 |
| RV/TLC, % | 59.45 ±8.17 | 61.18 ±8.64 | 0.086 |
| Emphysema destruction severity, LAA -950HU | 35.33 ±8.44 | 35.00 ±8.71 | 0.754 |
| Pi10 | 2.61 ±0.30 | 2.64 ±0.30 | 0.513 |
| Chronic kidney disease | 2 (2.6) | 14 (4.1) | 0.767 |
| Congestive heart failure | 1 (1.3) | 9 (2.6) | 0.777 |
| Pulmonary Arterial Hypertension | 8 (10.3) | 24 (7.0) | 0.448 |
| Atherosclerosis | 9 (11.5) | 79 (22.9) | **0.038** |
| Myocardial Infarction | 2 (2.6) | 23 (6.7) | 0.262 |
| Coronary Artery disease | 5 (6.4) | 41 (11.9) | 0.230 |
| Cerebrovasculair accident | 1 (1.3) | 13 (3.8) | 0.448 |
| Malignancy | 15 (19.2) | 59 (17.1) | 0.778 |
| Autoimmune disorder | 3 (3.8) | 25 (7.2) | 0.402 |
| Hypertension | 26 (33.3) | 118 (34.2) | 0.989 |
| Diabetes Mellitus | 5 (6.4) | 17 (4.9) | 0.802 |
| CRP, mg/L | 3.00 ±3.42 | 3.45 ±3.76 | 0.305 |

## Supplementary Table S3.6: Baseline characteristics by Hemoglobin concentration as measured in the RBC/PLT channel (HGB)

|  | **Above reference interval** | **Within normal reference interval** | **p** |
| --- | --- | --- | --- |
| n | 53 | 428 |  |
| Age, years | 62.58 ±6.70 | 61.47 ±7.37 | 0.293 |
| Sex, female | 44 (83.0) | 297 (69.4) | 0.057 |
| BMI, kg/m2 | 23.84 ±4.23 | 24.37 ±4.20 | 0.390 |
| Smoking status |  |  | 0.018 |
| Current smoker | 1 (1.9) | 7 (1.6) |  |
| Ex-smoker | 51 (96.2) | 419 (98.4) |  |
| Never smoker | 1 (1.9) | 0 (0.0) |  |
| Packyears | 38.79 ±20.43 | 40.74 ±19.17 | 0.493 |
| Use of ICS or ICS/LABA | 40 (93.0) | 366 (95.3) | 0.775 |
| Number of exacerbations in the previous year | 1.00 [0.00, 2.00] | 2.00 [1.00, 3.00] | 0.306 |
| Hospitalization due to an exacerbation in the previous year |  |  | 0.363 |
| Yes | 16 (84.2) | 164 (74.9) |  |
| No | 2 (10.5) | 50 (22.8) |  |
| Unknown | 1 (5.3) | 5 (2.3) |  |
| Number of hospitalizations in the previous year | 0.39 ±0.81 | 0.48 ±1.08 | 0.582 |
| CAT total score | 20.73 ±5.44 | 22.02 ±6.08 | 0.148 |
| CCQ total score | 2.88 ±0.66 | 3.04 ±0.92 | 0.246 |
| mMRC | 3.00 [2.00, 3.00] | 3.00 [2.00, 3.00] | 0.397 |
| SGRQ total score | 54.16 ±11.87 | 57.59 ±13.19 | 0.077 |
| FEV1 % predicted | 29.07 ±11.38 | 29.06 ±9.51 | 0.995 |
| RV/TLC, % | 61.70 ±9.48 | 60.68 ±8.47 | 0.415 |
| Emphysema destruction severity, LAA -950HU | 37.67 ±7.26 | 34.86 ±8.84 | 0.028 |
| Pi10 | 2.61 ±0.29 | 2.63 ±0.30 | 0.663 |
| Chronic kidney disease | 0 (0.0) | 14 (3.7) | 0.359 |
| Congestive heart failure | 2 (4.3) | 9 (2.4) | 0.792 |
| Pulmonary Arterial Hypertension | 4 (8.5) | 27 (7.2) | 0.981 |
| Atherosclerosis | 5 (10.6) | 76 (20.3) | 0.164 |
| Myocardial Infarction | 2 (4.3) | 22 (5.9) | 0.905 |
| Coronary Artery disease | 2 (4.3) | 42 (11.2) | 0.222 |
| Cerebrovasculair accident | 1 (2.1) | 11 (2.9) | 1.000 |
| Malignancy | 8 (17.0) | 66 (17.6) | 1.000 |
| Autoimmune disorder | 5 (10.6) | 22 (5.9) | 0.348 |
| Hypertension | 12 (25.5) | 131 (35.0) | 0.258 |
| Diabetes Mellitus | 0 (0.0) | 21 (5.6) | 0.190 |
| CRP, mg/L | 2.99 ±2.92 | 3.33 ±3.70 | 0.520 |
| Data are presented as n, n (%), mean±SD or median [interquartile range], unless otherwise specified. Univariate differences were tested with Chi-square tests, independent T-tests and Mann-whitney U-tests as appropriate. BMI: Body Mass Index; ICS: inhaled corticosteroids; LABA: long acting β2 agonist; CAT: COPD assessment test; CCQ: Clinical COPD questionnaire; CCQ: Clinical COPD Questionnaire; mMRC: Modified Medical Research Council Dyspnea Scale; SGRQ: St. George’s Respiratory Questionnaire; FEV_1_: Forced Expiratory Volume in 1 second; RV/TLC: Residual Volume/Total Lung Capacity; LAA: low attenuation areas < −950 hounsfield units (HU) on the inspiratory CT scan; Pi10: 10-mm internal luminal perimeter; CRP: C-Reactive Protein. | | | |

## Supplementary Table S3.7: Baseline characteristics by Immature granulocytes (IG)

|  | **Above reference interval** | **Within normal reference interval** | **p** |
| --- | --- | --- | --- |
| n | 49 | 446 |  |
| Age, years | 62.10 ±7.30 | 61.64 ±7.44 | 0.680 |
| Sex, female | 35 (71.4) | 310 (69.5) | 0.909 |
| BMI, kg/m2 | 24.69 ±4.26 | 24.24 ±4.23 | 0.475 |
| Smoking status |  |  | 0.940 |
| Current smoker | 1 (2.0) | 8 (1.8) |  |
| Ex-smoker | 48 (98.0) | 435 (98.0) |  |
| Never smoker | 0 (0.0) | 1 (0.2) |  |
| Packyears | 40.87 ±21.46 | 40.83 ±19.25 | 0.989 |
| Use of ICS or ICS/LABA | 45 (100.0) | 372 (94.2) | 0.190 |
| Number of exacerbations in the previous year | 2.00 [2.00, 4.00] | 1.00 [1.00, 3.00] | **<0.001** |
| Hospitalization due to an exacerbation in the previous year |  |  | 0.728 |
| Yes | 20 (80.0) | 169 (76.1) |  |
| No | 5 (20.0) | 48 (21.6) |  |
| Unknown | 0 (0.0) | 5 (2.3) |  |
| Number of hospitalizations in the previous year | 0.65 ±1.20 | 0.45 ±1.03 | 0.217 |
| CAT total score | 21.10 ±6.85 | 21.90 ±5.87 | 0.380 |
| CCQ total score | 2.99 ±0.98 | 3.02 ±0.89 | 0.809 |
| mMRC | 2.00 [2.00, 3.00] | 3.00 [2.00, 3.00] | 0.120 |
| SGRQ total score | 58.56 ±15.08 | 57.09 ±12.87 | 0.458 |
| FEV1 % predicted | 29.18 ±8.34 | 28.99 ±9.91 | 0.897 |
| RV/TLC, % | 60.82 ±7.69 | 60.82 ±8.73 | 1.000 |
| Emphysema destruction severity, LAA -950HU | 35.22 ±7.86 | 35.07 ±8.85 | 0.915 |
| Pi10 | 2.63 ±0.27 | 2.63 ±0.30 | 0.904 |
| Chronic kidney disease | 1 (2.4) | 15 (3.8) | 0.987 |
| Congestive heart failure | 0 (0.0) | 11 (2.8) | 0.571 |
| Pulmonary Arterial Hypertension | 1 (2.4) | 31 (7.9) | 0.335 |
| Atherosclerosis | 9 (22.0) | 79 (20.2) | 0.952 |
| Myocardial Infarction | 4 (9.8) | 22 (5.6) | 0.476 |
| Coronary Artery disease | 4 (9.8) | 43 (11.0) | 1.000 |
| Cerebrovasculair accident | 1 (2.4) | 13 (3.3) | 1.000 |
| Malignancy | 6 (14.6) | 69 (17.6) | 0.789 |
| Autoimmune disorder | 2 (4.9) | 26 (6.6) | 0.916 |
| Hypertension | 11 (26.8) | 136 (34.8) | 0.396 |
| Diabetes Mellitus | 1 (2.4) | 21 (5.4) | 0.661 |
| CRP, mg/L | 3.57 ±3.96 | 3.30 ±3.63 | 0.625 |
| Data are presented as n, n (%), mean±SD or median [interquartile range], unless otherwise specified. Univariate differences were tested with Chi-square tests, independent T-tests and Mann-whitney U-tests as appropriate. BMI: Body Mass Index; ICS: inhaled corticosteroids; LABA: long acting β2 agonist; CAT: COPD assessment test; CCQ: Clinical COPD questionnaire; CCQ: Clinical COPD Questionnaire; mMRC: Modified Medical Research Council Dyspnea Scale; SGRQ: St. George’s Respiratory Questionnaire; FEV_1_: Forced Expiratory Volume in 1 second; RV/TLC: Residual Volume/Total Lung Capacity; LAA: low attenuation areas < −950 hounsfield units (HU) on the inspiratory CT scan; Pi10: 10-mm internal luminal perimeter; CRP: C-Reactive Protein. | | | |

## Supplementary Table S3.8: Baseline characteristics by Lymphocytes (absolute count) (LYMPH)

|  | **Below reference interval** | **Within normal reference interval** | **p** |
| --- | --- | --- | --- |
| n | 39 | 426 |  |
| Age, years | 64.95 ±7.58 | 61.48 ±7.38 | **0.005** |
| Sex, female | 18 (46.2) | 301 (70.7) | **0.003** |
| BMI, kg/m2 | 24.47 ±4.00 | 24.18 ±4.25 | 0.682 |
| Smoking status |  |  | 0.915 |
| Current smoker | 1 (2.6) | 8 (1.9) |  |
| Ex-smoker | 38 (97.4) | 415 (97.9) |  |
| Never smoker | 0 (0.0) | 1 (0.2) |  |
| Packyears | 39.25 ±14.86 | 40.67 ±19.67 | 0.665 |
| Use of ICS or ICS/LABA | 32 (91.4) | 360 (94.7) | 0.665 |
| Number of exacerbations in the previous year | 2.00 [1.00, 3.25] | 1.00 [1.00, 3.00] | 0.053 |
| Hospitalization due to an exacerbation in the previous year |  |  | 0.461 |
| Yes | 25 (83.3) | 151 (75.1) |  |
| No | 4 (13.3) | 46 (22.9) |  |
| Unknown | 1 (3.3) | 4 (2.0) |  |
| Number of hospitalizations in the previous year | 0.86 ±1.44 | 0.43 ±1.00 | **0.020** |
| CAT total score | 21.58 ±5.62 | 21.72 ±5.97 | 0.885 |
| CCQ total score | 2.91 ±0.86 | 3.01 ±0.90 | 0.516 |
| mMRC | 3.00 [2.00, 3.00] | 3.00 [2.00, 3.00] | 0.067 |
| SGRQ total score | 58.23 ±13.56 | 56.89 ±13.04 | 0.546 |
| FEV1 % predicted | 27.15 ±8.40 | 29.14 ±9.78 | 0.218 |
| RV/TLC, % | 61.90 ±8.29 | 60.73 ±8.69 | 0.421 |
| Emphysema destruction severity, LAA -950HU | 36.89 ±7.93 | 34.98 ±8.77 | 0.208 |
| Pi10 | 2.66 ±0.28 | 2.63 ±0.30 | 0.495 |
| Chronic kidney disease | 1 (3.2) | 15 (4.0) | 1.000 |
| Congestive heart failure | 0 (0.0) | 11 (2.9) | 0.700 |
| Pulmonary Arterial Hypertension | 0 (0.0) | 30 (7.9) | 0.204 |
| Atherosclerosis | 6 (19.4) | 78 (20.6) | 1.000 |
| Myocardial Infarction | 4 (12.9) | 20 (5.3) | 0.181 |
| Coronary Artery disease | 4 (12.9) | 40 (10.6) | 0.921 |
| Cerebrovasculair accident | 1 (3.2) | 13 (3.4) | 1.000 |
| Malignancy | 6 (19.4) | 65 (17.2) | 0.953 |
| Autoimmune disorder | 4 (12.9) | 23 (6.1) | 0.274 |
| Hypertension | 11 (35.5) | 126 (33.3) | 0.963 |
| Diabetes Mellitus | 0 (0.0) | 20 (5.3) | 0.379 |
| CRP, mg/L | 3.28 ±3.64 | 3.33 ±3.61 | 0.937 |
| Data are presented as n, n (%), mean±SD or median [interquartile range], unless otherwise specified. Univariate differences were tested with Chi-square tests, independent T-tests and Mann-whitney U-tests as appropriate. BMI: Body Mass Index; ICS: inhaled corticosteroids; LABA: long acting β2 agonist; CAT: COPD assessment test; CCQ: Clinical COPD questionnaire; CCQ: Clinical COPD Questionnaire; mMRC: Modified Medical Research Council Dyspnea Scale; SGRQ: St. George’s Respiratory Questionnaire; FEV_1_: Forced Expiratory Volume in 1 second; RV/TLC: Residual Volume/Total Lung Capacity; LAA: low attenuation areas < −950 hounsfield units (HU) on the inspiratory CT scan; Pi10: 10-mm internal luminal perimeter; CRP: C-Reactive Protein. | | | |

## Supplementary Table S3.9: Baseline characteristics by Lymphocytes (absolute count)

|  | **Above reference interval** | **Within normal reference interval** | **p** |
| --- | --- | --- | --- |
| n | 30 | 426 |  |
| Age, years | 60.37 ±6.97 | 61.48 ±7.38 | 0.423 |
| Sex, female | 26 (86.7) | 301 (70.7) | 0.095 |
| BMI, kg/m2 | 25.57 ±4.20 | 24.18 ±4.25 | 0.083 |
| Smoking status |  |  | 0.723 |
| Current smoker | 0 (0.0) | 8 (1.9) |  |
| Ex-smoker | 30 (100.0) | 415 (97.9) |  |
| Never smoker | 0 (0.0) | 1 (0.2) |  |
| Packyears | 45.07 ±21.61 | 40.67 ±19.67 | 0.241 |
| Use of ICS or ICS/LABA | 25 (100.0) | 360 (94.7) | 0.484 |
| Number of exacerbations in the previous year | 3.00 [1.00, 4.00] | 1.00 [1.00, 3.00] | **0.002** |
| Hospitalization due to an exacerbation in the previous year |  |  | 0.777 |
| Yes | 13 (81.2) | 151 (75.1) |  |
| No | 3 (18.8) | 46 (22.9) |  |
| Unknown | 0 (0.0) | 4 (2.0) |  |
| Number of hospitalizations in the previous year | 0.61 ±0.99 | 0.43 ±1.00 | 0.364 |
| CAT total score | 23.37 ±6.44 | 21.72 ±5.97 | 0.147 |
| CCQ total score | 3.22 ±0.85 | 3.01 ±0.90 | 0.229 |
| mMRC | 3.00 [2.00, 3.00] | 3.00 [2.00, 3.00] | 0.483 |
| SGRQ total score | 60.83 ±13.12 | 56.89 ±13.04 | 0.110 |
| FEV1 % predicted | 29.52 ±11.15 | 29.14 ±9.78 | 0.840 |
| RV/TLC, % | 60.69 ±8.34 | 60.73 ±8.69 | 0.980 |
| Emphysema destruction severity, LAA -950HU | 34.34 ±9.38 | 34.98 ±8.77 | 0.708 |
| Pi10 | 2.65 ±0.32 | 2.63 ±0.30 | 0.676 |
| Chronic kidney disease | 0 (0.0) | 15 (4.0) | 0.683 |
| Congestive heart failure | 0 (0.0) | 11 (2.9) | 0.863 |
| Pulmonary Arterial Hypertension | 2 (8.7) | 30 (7.9) | 1.000 |
| Atherosclerosis | 4 (17.4) | 78 (20.6) | 0.914 |
| Myocardial Infarction | 2 (8.7) | 20 (5.3) | 0.822 |
| Coronary Artery disease | 3 (13.0) | 40 (10.6) | 0.981 |
| Cerebrovasculair accident | 0 (0.0) | 13 (3.4) | 0.766 |
| Malignancy | 4 (17.4) | 65 (17.2) | 1.000 |
| Autoimmune disorder | 1 (4.3) | 23 (6.1) | 1.000 |
| Hypertension | 10 (43.5) | 126 (33.3) | 0.441 |
| Diabetes Mellitus | 2 (8.7) | 20 (5.3) | 0.822 |
| CRP, mg/L | 3.38 ±4.47 | 3.33 ±3.61 | 0.939 |
| Data are presented as n, n (%), mean±SD or median [interquartile range], unless otherwise specified. Univariate differences were tested with Chi-square tests, independent T-tests and Mann-whitney U-tests as appropriate. BMI: Body Mass Index; ICS: inhaled corticosteroids; LABA: long acting β2 agonist; CAT: COPD assessment test; CCQ: Clinical COPD questionnaire; CCQ: Clinical COPD Questionnaire; mMRC: Modified Medical Research Council Dyspnea Scale; SGRQ: St. George’s Respiratory Questionnaire; FEV_1_: Forced Expiratory Volume in 1 second; RV/TLC: Residual Volume/Total Lung Capacity; LAA: low attenuation areas < −950 hounsfield units (HU) on the inspiratory CT scan; Pi10: 10-mm internal luminal perimeter; CRP: C-Reactive Protein. | | | |

## Supplementary Table S3.10: Baseline characteristics by Side scattered light distribution width of de lymphocyte population (LY-X)

|  | **Below reference interval** | **Within normal reference interval** | **p** |
| --- | --- | --- | --- |
| n | 25 | 328 |  |
| Age, years | 60.52 ±9.76 | 61.42 ±7.04 | 0.549 |
| Sex, female | 20 (80.0) | 243 (74.1) | 0.677 |
| BMI, kg/m2 | 22.75 ±4.31 | 24.33 ±4.33 | 0.079 |
| Smoking status |  |  | **0.040** |
| Current smoker | 2 (8.0) | 4 (1.2) |  |
| Ex-smoker | 23 (92.0) | 322 (98.5) |  |
| Never smoker | 0 (0.0) | 1 (0.3) |  |
| Packyears | 34.48 ±13.82 | 41.14 ±19.73 | 0.098 |
| Use of ICS or ICS/LABA | 22 (100.0) | 277 (96.5) | 0.791 |
| Number of exacerbations in the previous year | 2.00 [0.00, 3.25] | 1.00 [1.00, 3.00] | 0.689 |
| Hospitalization due to an exacerbation in the previous year |  |  | 0.453 |
| Yes | 7 (63.6) | 117 (78.0) |  |
| No | 4 (36.4) | 31 (20.7) |  |
| Unknown | 0 (0.0) | 2 (1.3) |  |
| Number of hospitalizations in the previous year | 0.52 ±1.31 | 0.43 ±1.00 | 0.668 |
| CAT total score | 21.46 ±5.93 | 21.65 ±5.84 | 0.879 |
| CCQ total score | 3.06 ±0.98 | 2.96 ±0.87 | 0.614 |
| mMRC | 3.00 [2.00, 3.00] | 3.00 [2.00, 3.00] | 0.048 |
| SGRQ total score | 57.57 ±13.51 | 56.93 ±12.55 | 0.812 |
| FEV1 % predicted | 30.01 ±9.33 | 29.05 ±9.87 | 0.638 |
| RV/TLC, % | 60.09 ±8.68 | 61.04 ±8.54 | 0.590 |
| Emphysema destruction severity, LAA -950HU | 37.92 ±6.71 | 35.15 ±8.97 | 0.140 |
| Pi10 | 2.64 ±0.22 | 2.62 ±0.30 | 0.838 |
| Chronic kidney disease | 1 (4.3) | 13 (4.5) | 1.000 |
| Congestive heart failure | 1 (4.3) | 7 (2.4) | 1.000 |
| Pulmonary Arterial Hypertension | 2 (8.7) | 20 (6.9) | 1.000 |
| Atherosclerosis | 5 (21.7) | 55 (19.0) | 0.960 |
| Myocardial Infarction | 1 (4.3) | 18 (6.2) | 1.000 |
| Coronary Artery disease | 2 (8.7) | 32 (11.0) | 1.000 |
| Cerebrovasculair accident | 2 (8.7) | 10 (3.4) | 0.486 |
| Malignancy | 2 (8.7) | 52 (17.9) | 0.400 |
| Autoimmune disorder | 3 (13.0) | 17 (5.9) | 0.361 |
| Hypertension | 10 (43.5) | 98 (33.8) | 0.476 |
| Diabetes Mellitus | 1 (4.3) | 13 (4.5) | 1.000 |
| CRP, mg/L | 2.62 ±3.78 | 3.35 ±3.85 | 0.361 |
| Data are presented as n, n (%), mean±SD or median [interquartile range], unless otherwise specified. Univariate differences were tested with Chi-square tests, independent T-tests and Mann-whitney U-tests as appropriate. BMI: Body Mass Index; ICS: inhaled corticosteroids; LABA: long acting β2 agonist; CAT: COPD assessment test; CCQ: Clinical COPD questionnaire; CCQ: Clinical COPD Questionnaire; mMRC: Modified Medical Research Council Dyspnea Scale; SGRQ: St. George’s Respiratory Questionnaire; FEV_1_: Forced Expiratory Volume in 1 second; RV/TLC: Residual Volume/Total Lung Capacity; LAA: low attenuation areas < −950 hounsfield units (HU) on the inspiratory CT scan; Pi10: 10-mm internal luminal perimeter; CRP: C-Reactive Protein. | | | |

## Supplementary Table S3.11: Baseline characteristics by Side scattered light distribution width of de lymphocyte population (LY-X)

|  | **Above reference interval** | **Within normal reference interval** | **p** |
| --- | --- | --- | --- |
| n | 142 | 328 |  |
| Age, years | 62.50 ±7.79 | 61.42 ±7.04 | 0.142 |
| Sex, female | 82 (57.7) | 243 (74.1) | **0.001** |
| BMI, kg/m2 | 24.46 ±3.95 | 24.33 ±4.33 | 0.758 |
| Smoking status |  |  | 0.615 |
| Current smoker | 3 (2.1) | 4 (1.2) |  |
| Ex-smoker | 138 (97.9) | 322 (98.5) |  |
| Never smoker | 0 (0.0) | 1 (0.3) |  |
| Packyears | 41.23 ±19.61 | 41.14 ±19.73 | 0.964 |
| Use of ICS or ICS/LABA | 118 (90.1) | 277 (96.5) | **0.014** |
| Number of exacerbations in the previous year | 2.00 [1.00, 3.00] | 1.00 [1.00, 3.00] | 0.216 |
| Hospitalization due to an exacerbation in the previous year |  |  | 0.538 |
| Yes | 65 (75.6) | 117 (78.0) |  |
| No | 18 (20.9) | 31 (20.7) |  |
| Unknown | 3 (3.5) | 2 (1.3) |  |
| Number of hospitalizations in the previous year | 0.57 ±1.09 | 0.43 ±1.00 | 0.187 |
| CAT total score | 22.26 ±6.28 | 21.65 ±5.84 | 0.316 |
| CCQ total score | 3.13 ±0.92 | 2.96 ±0.87 | 0.072 |
| mMRC | 3.00 [2.00, 3.00] | 3.00 [2.00, 3.00] | 0.444 |
| SGRQ total score | 57.88 ±14.25 | 56.93 ±12.55 | 0.477 |
| FEV1 % predicted | 28.74 ±9.64 | 29.05 ±9.87 | 0.749 |
| RV/TLC, % | 60.45 ±8.86 | 61.04 ±8.54 | 0.493 |
| Emphysema destruction severity, LAA -950HU | 34.43 ±8.47 | 35.15 ±8.97 | 0.430 |
| Pi10 | 2.66 ±0.30 | 2.62 ±0.30 | 0.288 |
| Chronic kidney disease | 2 (1.7) | 13 (4.5) | 0.280 |
| Congestive heart failure | 3 (2.5) | 7 (2.4) | 1.000 |
| Pulmonary Arterial Hypertension | 10 (8.4) | 20 (6.9) | 0.747 |
| Atherosclerosis | 28 (23.5) | 55 (19.0) | 0.364 |
| Myocardial Infarction | 7 (5.9) | 18 (6.2) | 1.000 |
| Coronary Artery disease | 13 (10.9) | 32 (11.0) | 1.000 |
| Cerebrovasculair accident | 2 (1.7) | 10 (3.4) | 0.522 |
| Malignancy | 21 (17.6) | 52 (17.9) | 1.000 |
| Autoimmune disorder | 8 (6.7) | 17 (5.9) | 0.918 |
| Hypertension | 39 (32.8) | 98 (33.8) | 0.934 |
| Diabetes Mellitus | 8 (6.7) | 13 (4.5) | 0.493 |
| CRP, mg/L | 3.39 ±3.19 | 3.35 ±3.85 | 0.923 |
| Data are presented as n, n (%), mean±SD or median [interquartile range], unless otherwise specified. Univariate differences were tested with Chi-square tests, independent T-tests and Mann-whitney U-tests as appropriate. BMI: Body Mass Index; ICS: inhaled corticosteroids; LABA: long acting β2 agonist; CAT: COPD assessment test; CCQ: Clinical COPD questionnaire; CCQ: Clinical COPD Questionnaire; mMRC: Modified Medical Research Council Dyspnea Scale; SGRQ: St. George’s Respiratory Questionnaire; FEV_1_: Forced Expiratory Volume in 1 second; RV/TLC: Residual Volume/Total Lung Capacity; LAA: low attenuation areas < −950 hounsfield units (HU) on the inspiratory CT scan; Pi10: 10-mm internal luminal perimeter; CRP: C-Reactive Protein. | | | |

## Supplementary Table S3.12: Baseline characteristics by Fluorescent light distribution width of the lymphocyte population (LY-Y)

|  | **Above reference interval** | **Within normal reference interval** | **p** |
| --- | --- | --- | --- |
| n | 170 | 324 |  |
| Age, years | 61.13 ±6.88 | 61.98 ±7.70 | 0.228 |
| Sex, female | 121 (71.2) | 223 (68.8) | 0.662 |
| BMI, kg/m2 | 24.69 ±4.61 | 24.05 ±4.00 | 0.107 |
| Smoking status |  |  | 0.631 |
| Current smoker | 4 (2.4) | 5 (1.6) |  |
| Ex-smoker | 166 (97.6) | 316 (98.1) |  |
| Never smoker | 0 (0.0) | 1 (0.3) |  |
| Packyears | 41.19 ±19.44 | 40.68 ±19.52 | 0.782 |
| Use of ICS or ICS/LABA | 150 (98.0) | 266 (93.0) | **0.042** |
| Number of exacerbations in the previous year | 2.00 [1.00, 3.00] | 1.00 [1.00, 3.00] | 0.060 |
| Hospitalization due to an exacerbation in the previous year |  |  | 0.651 |
| Yes | 75 (78.1) | 113 (75.3) |  |
| No | 20 (20.8) | 33 (22.0) |  |
| Unknown | 1 (1.0) | 4 (2.7) |  |
| Number of hospitalizations in the previous year | 0.49 ±0.99 | 0.46 ±1.07 | 0.761 |
| CAT total score | 21.43 ±5.69 | 22.02 ±6.13 | 0.303 |
| CCQ total score | 3.01 ±0.86 | 3.02 ±0.92 | 0.872 |
| mMRC | 3.00 [2.00, 3.00] | 3.00 [2.00, 3.00] | 0.148 |
| SGRQ total score | 57.18 ±12.10 | 57.29 ±13.64 | 0.930 |
| FEV1 % predicted | 29.50 ±9.65 | 28.78 ±9.82 | 0.439 |
| RV/TLC, % | 60.49 ±8.35 | 60.99 ±8.79 | 0.543 |
| Emphysema destruction severity, LAA -950HU | 34.90 ±8.88 | 35.19 ±8.70 | 0.731 |
| Pi10 | 2.64 ±0.31 | 2.63 ±0.30 | 0.599 |
| Chronic kidney disease | 6 (4.0) | 10 (3.6) | 1.000 |
| Congestive heart failure | 4 (2.7) | 7 (2.5) | 1.000 |
| Pulmonary Arterial Hypertension | 16 (10.7) | 16 (5.7) | 0.092 |
| Atherosclerosis | 32 (21.3) | 56 (19.9) | 0.827 |
| Myocardial Infarction | 8 (5.3) | 18 (6.4) | 0.816 |
| Coronary Artery disease | 16 (10.7) | 31 (11.0) | 1.000 |
| Cerebrovasculair accident | 5 (3.3) | 9 (3.2) | 1.000 |
| Malignancy | 28 (18.7) | 47 (16.7) | 0.709 |
| Autoimmune disorder | 12 (8.0) | 16 (5.7) | 0.471 |
| Hypertension | 52 (34.7) | 94 (33.5) | 0.883 |
| Diabetes Mellitus | 9 (6.0) | 13 (4.6) | 0.698 |
| CRP, mg/L | 3.44 ±3.35 | 3.22 ±3.72 | 0.505 |
| Data are presented as n, n (%), mean±SD or median [interquartile range], unless otherwise specified. Univariate differences were tested with Chi-square tests, independent T-tests and Mann-whitney U-tests as appropriate. BMI: Body Mass Index; ICS: inhaled corticosteroids; LABA: long acting β2 agonist; CAT: COPD assessment test; CCQ: Clinical COPD questionnaire; CCQ: Clinical COPD Questionnaire; mMRC: Modified Medical Research Council Dyspnea Scale; SGRQ: St. George’s Respiratory Questionnaire; FEV_1_: Forced Expiratory Volume in 1 second; RV/TLC: Residual Volume/Total Lung Capacity; LAA: low attenuation areas < −950 hounsfield units (HU) on the inspiratory CT scan; Pi10: 10-mm internal luminal perimeter; CRP: C-Reactive Protein. | | | |

## Supplementary Table S3.13: Baseline characteristics by Forward scattered light distribution width of the lymphocyte population (LY-Z)

|  | **Below reference interval** | **Within normal reference interval** | **p** |
| --- | --- | --- | --- |
| n | 317 | 170 |  |
| Age, years | 61.70 ±7.47 | 61.84 ±7.28 | 0.851 |
| Sex, female | 215 (67.8) | 124 (72.9) | 0.286 |
| BMI, kg/m2 | 24.09 ±4.11 | 24.67 ±4.41 | 0.148 |
| Smoking status |  |  | 0.761 |
| Current smoker | 6 (1.9) | 3 (1.8) |  |
| Ex-smoker | 309 (97.8) | 166 (98.2) |  |
| Never smoker | 1 (0.3) | 0 (0.0) |  |
| Packyears | 42.39 ±20.69 | 38.16 ±17.02 | **0.024** |
| Use of ICS or ICS/LABA | 271 (94.8) | 140 (94.6) | 1.000 |
| Number of exacerbations in the previous year | 2.00 [1.00, 3.00] | 2.00 [1.00, 3.00] | 0.334 |
| Hospitalization due to an exacerbation in the previous year |  |  | **0.020** |
| Yes | 102 (83.6) | 83 (70.9) |  |
| No | 20 (16.4) | 30 (25.6) |  |
| Unknown | 0 (0.0) | 4 (3.4) |  |
| Number of hospitalizations in the previous year | 0.46 ±1.09 | 0.49 ±0.97 | 0.798 |
| CAT total score | 21.80 ±5.81 | 22.07 ±6.21 | 0.639 |
| CCQ total score | 3.03 ±0.90 | 3.02 ±0.89 | 0.920 |
| mMRC | 3.00 [2.00, 3.00] | 3.00 [2.00, 3.00] | 0.040 |
| SGRQ total score | 57.96 ±12.99 | 56.34 ±13.10 | 0.199 |
| FEV1 % predicted | 28.09 ±8.89 | 30.64 ±10.73 | **0.005** |
| RV/TLC, % | 61.31 ±8.49 | 59.93 ±8.84 | 0.094 |
| Emphysema destruction severity, LAA -950HU | 34.94 ±8.90 | 35.18 ±8.56 | 0.787 |
| Pi10 | 2.63 ±0.30 | 2.64 ±0.31 | 0.835 |
| Chronic kidney disease | 13 (4.5) | 3 (2.1) | 0.343 |
| Congestive heart failure | 6 (2.1) | 5 (3.6) | 0.561 |
| Pulmonary Arterial Hypertension | 20 (7.0) | 11 (7.9) | 0.894 |
| Atherosclerosis | 56 (19.5) | 32 (22.9) | 0.500 |
| Myocardial Infarction | 19 (6.6) | 7 (5.0) | 0.659 |
| Coronary Artery disease | 32 (11.1) | 15 (10.7) | 1.000 |
| Cerebrovasculair accident | 11 (3.8) | 3 (2.1) | 0.528 |
| Malignancy | 45 (15.7) | 29 (20.7) | 0.248 |
| Autoimmune disorder | 22 (7.7) | 6 (4.3) | 0.264 |
| Hypertension | 97 (33.8) | 50 (35.7) | 0.777 |
| Diabetes Mellitus | 10 (3.5) | 12 (8.6) | **0.046** |
| CRP, mg/L | 3.45 ±3.93 | 3.11 ±3.16 | 0.333 |
| Data are presented as n, n (%), mean±SD or median [interquartile range], unless otherwise specified. Univariate differences were tested with Chi-square tests, independent T-tests and Mann-whitney U-tests as appropriate. BMI: Body Mass Index; ICS: inhaled corticosteroids; LABA: long acting β2 agonist; CAT: COPD assessment test; CCQ: Clinical COPD questionnaire; CCQ: Clinical COPD Questionnaire; mMRC: Modified Medical Research Council Dyspnea Scale; SGRQ: St. George’s Respiratory Questionnaire; FEV_1_: Forced Expiratory Volume in 1 second; RV/TLC: Residual Volume/Total Lung Capacity; LAA: low attenuation areas < −950 hounsfield units (HU) on the inspiratory CT scan; Pi10: 10-mm internal luminal perimeter; CRP: C-Reactive Protein. | | | |

## Supplementary Table S3.14: Baseline characteristics by Macrocytic red blood cells (MacroR)

|  | **Above reference interval** | **Within normal reference interval** | **p** |
| --- | --- | --- | --- |
| n | 66 | 431 |  |
| Age, years | 62.44 ±8.61 | 61.62 ±7.21 | 0.402 |
| Sex, female | 38 (57.6) | 306 (71.0) | **0.040** |
| BMI, kg/m2 | 24.41 ±4.23 | 24.26 ±4.22 | 0.787 |
| Smoking status |  |  | **0.018** |
| Current smoker | 4 (6.2) | 5 (1.2) |  |
| Ex-smoker | 61 (93.8) | 424 (98.6) |  |
| Never smoker | 0 (0.0) | 1 (0.2) |  |
| Packyears | 39.52 ±17.02 | 41.05 ±19.85 | 0.559 |
| Use of ICS or ICS/LABA | 55 (93.2) | 364 (95.0) | 0.787 |
| Number of exacerbations in the previous year | 2.00 [1.00, 2.00] | 2.00 [1.00, 3.00] | 0.790 |
| Hospitalization due to an exacerbation in the previous year |  |  | 0.838 |
| Yes | 27 (79.4) | 162 (75.7) |  |
| No | 6 (17.6) | 47 (22.0) |  |
| Unknown | 1 (2.9) | 5 (2.3) |  |
| Number of hospitalizations in the previous year | 0.37 ±0.80 | 0.48 ±1.06 | 0.439 |
| CAT total score | 22.22 ±6.19 | 21.80 ±5.98 | 0.605 |
| CCQ total score | 3.02 ±1.00 | 3.03 ±0.88 | 0.957 |
| mMRC | 3.00 [2.00, 3.00] | 3.00 [2.00, 3.00] | 0.280 |
| SGRQ total score | 56.27 ±15.25 | 57.48 ±12.77 | 0.492 |
| FEV1 % predicted | 28.96 ±9.73 | 28.93 ±9.71 | 0.984 |
| RV/TLC, % | 60.30 ±9.26 | 60.97 ±8.48 | 0.559 |
| Emphysema destruction severity, LAA -950HU | 35.48 ±7.60 | 35.00 ±8.91 | 0.692 |
| Pi10 | 2.60 ±0.30 | 2.64 ±0.30 | 0.295 |
| Chronic kidney disease | 2 (3.6) | 14 (3.7) | 1.000 |
| Congestive heart failure | 5 (9.1) | 6 (1.6) | 0.004 |
| Pulmonary Arterial Hypertension | 2 (3.6) | 30 (7.9) | 0.393 |
| Atherosclerosis | 15 (27.3) | 73 (19.2) | 0.226 |
| Myocardial Infarction | 8 (14.5) | 18 (4.7) | **0.010** |
| Coronary Artery disease | 10 (18.2) | 37 (9.7) | 0.098 |
| Cerebrovasculair accident | 2 (3.6) | 12 (3.2) | 1.000 |
| Malignancy | 10 (18.2) | 65 (17.1) | 0.995 |
| Autoimmune disorder | 3 (5.5) | 25 (6.6) | 0.981 |
| Hypertension | 18 (32.7) | 130 (34.2) | 0.948 |
| Diabetes Mellitus | 1 (1.8) | 21 (5.5) | 0.399 |
| CRP, mg/L | 3.12 ±3.14 | 3.36 ±3.73 | 0.626 |
| Data are presented as n, n (%), mean±SD or median [interquartile range], unless otherwise specified. Univariate differences were tested with Chi-square tests, independent T-tests and Mann-whitney U-tests as appropriate. BMI: Body Mass Index; ICS: inhaled corticosteroids; LABA: long acting β2 agonist; CAT: COPD assessment test; CCQ: Clinical COPD questionnaire; CCQ: Clinical COPD Questionnaire; mMRC: Modified Medical Research Council Dyspnea Scale; SGRQ: St. George’s Respiratory Questionnaire; FEV_1_: Forced Expiratory Volume in 1 second; RV/TLC: Residual Volume/Total Lung Capacity; LAA: low attenuation areas < −950 hounsfield units (HU) on the inspiratory CT scan; Pi10: 10-mm internal luminal perimeter; CRP: C-Reactive Protein. | | | |

## Supplementary Table S3.15: Baseline characteristics by Microcytic red blood cells (MicroR)

|  | **Above reference interval** | **Within normal reference interval** | **p** |
| --- | --- | --- | --- |
| n | 33 | 462 |  |
| Age, years | 62.24 ±8.80 | 61.65 ±7.32 | 0.660 |
| Sex, female | 22 (66.7) | 321 (69.5) | 0.886 |
| BMI, kg/m2 | 24.62 ±4.20 | 24.24 ±4.23 | 0.617 |
| Smoking status |  |  | 0.702 |
| Current smoker | 0 (0.0) | 9 (2.0) |  |
| Ex-smoker | 32 (100.0) | 451 (97.8) |  |
| Never smoker | 0 (0.0) | 1 (0.2) |  |
| Packyears | 44.12 ±23.48 | 40.63 ±19.23 | 0.328 |
| Use of ICS or ICS/LABA | 25 (92.6) | 392 (94.9) | 0.937 |
| Number of exacerbations in the previous year | 1.00 [0.75, 2.00] | 2.00 [1.00, 3.00] | 0.291 |
| Hospitalization due to an exacerbation in the previous year |  |  | 0.777 |
| Yes | 10 (83.3) | 178 (76.1) |  |
| No | 2 (16.7) | 50 (21.4) |  |
| Unknown | 0 (0.0) | 6 (2.6) |  |
| Number of hospitalizations in the previous year | 0.34 ±0.55 | 0.48 ±1.07 | 0.482 |
| CAT total score | 24.50 ±6.70 | 21.68 ±5.91 | **0.010** |
| CCQ total score | 3.28 ±1.01 | 3.01 ±0.89 | 0.109 |
| mMRC | 3.00 [2.00, 3.00] | 3.00 [2.00, 3.00] | 0.047 |
| SGRQ total score | 61.14 ±15.99 | 57.07 ±12.89 | 0.091 |
| FEV1 % predicted | 29.30 ±9.52 | 28.91 ±9.77 | 0.824 |
| RV/TLC, % | 61.44 ±9.18 | 60.86 ±8.57 | 0.708 |
| Emphysema destruction severity, LAA -950HU | 34.42 ±9.59 | 35.12 ±8.69 | 0.660 |
| Pi10 | 2.60 ±0.32 | 2.64 ±0.30 | 0.434 |
| Chronic kidney disease | 1 (3.2) | 15 (3.7) | 1.000 |
| Congestive heart failure | 1 (3.2) | 10 (2.5) | 1.000 |
| Pulmonary Arterial Hypertension | 5 (16.1) | 27 (6.7) | 0.117 |
| Atherosclerosis | 9 (29.0) | 78 (19.5) | 0.294 |
| Myocardial Infarction | 4 (12.9) | 22 (5.5) | 0.200 |
| Coronary Artery disease | 6 (19.4) | 41 (10.2) | 0.203 |
| Cerebrovasculair accident | 1 (3.2) | 13 (3.2) | 1.000 |
| Malignancy | 7 (22.6) | 68 (17.0) | 0.582 |
| Autoimmune disorder | 1 (3.2) | 27 (6.7) | 0.700 |
| Hypertension | 12 (38.7) | 135 (33.7) | 0.708 |
| Diabetes Mellitus | 3 (9.7) | 18 (4.5) | 0.389 |
| CRP, mg/L | 4.11 ±4.42 | 3.27 ±3.60 | 0.206 |
| Data are presented as n, n (%), mean±SD or median [interquartile range], unless otherwise specified. Univariate differences were tested with Chi-square tests, independent T-tests and Mann-whitney U-tests as appropriate. BMI: Body Mass Index; ICS: inhaled corticosteroids; LABA: long acting β2 agonist; CAT: COPD assessment test; CCQ: Clinical COPD questionnaire; CCQ: Clinical COPD Questionnaire; mMRC: Modified Medical Research Council Dyspnea Scale; SGRQ: St. George’s Respiratory Questionnaire; FEV_1_: Forced Expiratory Volume in 1 second; RV/TLC: Residual Volume/Total Lung Capacity; LAA: low attenuation areas < −950 hounsfield units (HU) on the inspiratory CT scan; Pi10: 10-mm internal luminal perimeter; CRP: C-Reactive Protein. | | | |

## Supplementary Table S3.16: Baseline characteristics by Monocytes (absolute count) (MONO)

|  | **Above reference interval** | **Within normal reference interval** | **p** |
| --- | --- | --- | --- |
| n | 108 | 375 |  |
| Age, years | 62.30 ±7.59 | 61.42 ±7.34 | 0.278 |
| Sex, female | 69 (63.9) | 268 (71.5) | 0.164 |
| BMI, kg/m2 | 24.45 ±4.41 | 24.22 ±4.17 | 0.617 |
| Smoking status |  |  | **0.048** |
| Current smoker | 0 (0.0) | 9 (2.4) |  |
| Ex-smoker | 107 (99.1) | 364 (97.6) |  |
| Never smoker | 1 (0.9) | 0 (0.0) |  |
| Packyears | 43.17 ±22.23 | 40.14 ±18.66 | 0.158 |
| Use of ICS or ICS/LABA | 93 (94.9) | 314 (94.6) | 1.000 |
| Number of exacerbations in the previous year | 2.00 [1.00, 2.50] | 2.00 [1.00, 3.00] | 0.391 |
| Hospitalization due to an exacerbation in the previous year |  |  | 0.417 |
| Yes | 40 (81.6) | 143 (75.3) |  |
| No | 9 (18.4) | 42 (22.1) |  |
| Unknown | 0 (0.0) | 5 (2.6) |  |
| Number of hospitalizations in the previous year | 0.40 ±0.74 | 0.49 ±1.12 | 0.473 |
| CAT total score | 21.59 ±6.23 | 21.81 ±5.92 | 0.735 |
| CCQ total score | 3.02 ±0.87 | 3.01 ±0.91 | 0.944 |
| mMRC | 3.00 [2.00, 3.00] | 3.00 [2.00, 3.00] | 0.671 |
| SGRQ total score | 56.94 ±13.40 | 57.21 ±13.02 | 0.852 |
| FEV1 % predicted | 28.79 ±8.88 | 29.01 ±9.92 | 0.835 |
| RV/TLC, % | 61.06 ±8.66 | 60.80 ±8.63 | 0.778 |
| Emphysema destruction severity, LAA -950HU | 33.95 ±8.07 | 35.36 ±8.96 | 0.149 |
| Pi10 | 2.65 ±0.31 | 2.63 ±0.30 | 0.527 |
| Chronic kidney disease | 6 (6.2) | 10 (3.1) | 0.258 |
| Congestive heart failure | 3 (3.1) | 8 (2.5) | 1.000 |
| Pulmonary Arterial Hypertension | 5 (5.2) | 27 (8.3) | 0.435 |
| Atherosclerosis | 22 (22.9) | 62 (19.0) | 0.487 |
| Myocardial Infarction | 9 (9.4) | 15 (4.6) | 0.127 |
| Coronary Artery disease | 15 (15.6) | 30 (9.2) | 0.109 |
| Cerebrovasculair accident | 4 (4.2) | 10 (3.1) | 0.838 |
| Malignancy | 14 (14.6) | 61 (18.7) | 0.437 |
| Autoimmune disorder | 5 (5.2) | 22 (6.7) | 0.761 |
| Hypertension | 36 (37.5) | 108 (33.1) | 0.502 |
| Diabetes Mellitus | 3 (3.1) | 19 (5.8) | 0.432 |
| CRP, mg/L | 3.97 ±4.14 | 3.18 ±3.53 | **0.048** |
| Data are presented as n, n (%), mean±SD or median [interquartile range], unless otherwise specified. Univariate differences were tested with Chi-square tests, independent T-tests and Mann-whitney U-tests as appropriate. BMI: Body Mass Index; ICS: inhaled corticosteroids; LABA: long acting β2 agonist; CAT: COPD assessment test; CCQ: Clinical COPD questionnaire; CCQ: Clinical COPD Questionnaire; mMRC: Modified Medical Research Council Dyspnea Scale; SGRQ: St. George’s Respiratory Questionnaire; FEV_1_: Forced Expiratory Volume in 1 second; RV/TLC: Residual Volume/Total Lung Capacity; LAA: low attenuation areas < −950 hounsfield units (HU) on the inspiratory CT scan; Pi10: 10-mm internal luminal perimeter; CRP: C-Reactive Protein. | | | |

## Supplementary Table S3.17: Baseline characteristics by Side scattered light distribution width of de monocyte population (MO-X)

|  | **Above reference interval** | **Within normal reference interval** | **p** |
| --- | --- | --- | --- |
| n | 22 | 387 |  |
| Age, years | 62.09 ±9.93 | 61.53 ±7.23 | 0.728 |
| Sex, female | 13 (59.1) | 273 (70.5) | 0.368 |
| BMI, kg/m2 | 24.92 ±4.62 | 24.33 ±4.21 | 0.520 |
| Smoking status |  |  | 0.792 |
| Current smoker | 0 (0.0) | 7 (1.8) |  |
| Ex-smoker | 22 (100.0) | 377 (97.9) |  |
| Never smoker | 0 (0.0) | 1 (0.3) |  |
| Packyears | 36.43 ±12.89 | 40.84 ±19.79 | 0.303 |
| Use of ICS or ICS/LABA | 17 (81.0) | 330 (94.6) | **0.041** |
| Number of exacerbations in the previous year | 1.00 [1.00, 2.00] | 2.00 [1.00, 3.00] | 0.649 |
| Hospitalization due to an exacerbation in the previous year |  |  | 0.171 |
| Yes | 12 (100.0) | 140 (76.9) |  |
| No | 0 (0.0) | 37 (20.3) |  |
| Unknown | 0 (0.0) | 5 (2.7) |  |
| Number of hospitalizations in the previous year | 0.64 ±1.05 | 0.46 ±1.06 | 0.436 |
| CAT total score | 23.18 ±6.76 | 21.50 ±6.00 | 0.206 |
| CCQ total score | 3.22 ±1.11 | 3.00 ±0.89 | 0.266 |
| mMRC | 3.00 [2.00, 3.00] | 3.00 [2.00, 3.00] | 0.919 |
| SGRQ total score | 56.00 ±16.07 | 57.03 ±13.18 | 0.724 |
| FEV1 % predicted | 28.14 ±9.10 | 28.97 ±9.81 | 0.698 |
| RV/TLC, % | 61.47 ±8.36 | 60.82 ±8.55 | 0.728 |
| Emphysema destruction severity, LAA -950HU | 31.09 ±9.09 | 34.82 ±9.03 | 0.061 |
| Pi10 | 2.71 ±0.33 | 2.64 ±0.30 | 0.274 |
| Chronic kidney disease | 1 (5.0) | 13 (3.9) | 1.000 |
| Congestive heart failure | 0 (0.0) | 8 (2.4) | 1.000 |
| Pulmonary Arterial Hypertension | 1 (5.0) | 25 (7.4) | 1.000 |
| Atherosclerosis | 8 (40.0) | 58 (17.2) | **0.024** |
| Myocardial Infarction | 1 (5.0) | 15 (4.5) | 1.000 |
| Coronary Artery disease | 4 (20.0) | 28 (8.3) | 0.169 |
| Cerebrovasculair accident | 0 (0.0) | 9 (2.7) | 0.995 |
| Malignancy | 5 (25.0) | 60 (17.8) | 0.609 |
| Autoimmune disorder | 2 (10.0) | 21 (6.2) | 0.843 |
| Hypertension | 8 (40.0) | 109 (32.3) | 0.643 |
| Diabetes Mellitus | 1 (5.0) | 17 (5.0) | 1.000 |
| CRP, mg/L | 4.45 ±3.43 | 3.43 ±3.80 | 0.220 |
| Data are presented as n, n (%), mean±SD or median [interquartile range], unless otherwise specified. Univariate differences were tested with Chi-square tests, independent T-tests and Mann-whitney U-tests as appropriate. BMI: Body Mass Index; ICS: inhaled corticosteroids; LABA: long acting β2 agonist; CAT: COPD assessment test; CCQ: Clinical COPD questionnaire; CCQ: Clinical COPD Questionnaire; mMRC: Modified Medical Research Council Dyspnea Scale; SGRQ: St. George’s Respiratory Questionnaire; FEV_1_: Forced Expiratory Volume in 1 second; RV/TLC: Residual Volume/Total Lung Capacity; LAA: low attenuation areas < −950 hounsfield units (HU) on the inspiratory CT scan; Pi10: 10-mm internal luminal perimeter; CRP: C-Reactive Protein. | | | |

## Supplementary Table S3.18: Baseline characteristics by Side scattered light distribution width of de monocyte population (MO-X)

|  | **Below reference interval** | **Within normal reference interval** | **p** |
| --- | --- | --- | --- |
| n | 86 | 387 |  |
| Age, years | 62.30 ±7.60 | 61.53 ±7.23 | 0.373 |
| Sex, female | 59 (68.6) | 273 (70.5) | 0.822 |
| BMI, kg/m2 | 23.93 ±4.25 | 24.33 ±4.21 | 0.430 |
| Smoking status |  |  | 0.853 |
| Current smoker | 2 (2.3) | 7 (1.8) |  |
| Ex-smoker | 84 (97.7) | 377 (97.9) |  |
| Never smoker | 0 (0.0) | 1 (0.3) |  |
| Packyears | 41.92 ±19.40 | 40.84 ±19.79 | 0.649 |
| Use of ICS or ICS/LABA | 70 (100.0) | 330 (94.6) | 0.092 |
| Number of exacerbations in the previous year | 2.00 [1.00, 3.00] | 2.00 [1.00, 3.00] | 0.367 |
| Hospitalization due to an exacerbation in the previous year |  |  | 0.174 |
| Yes | 37 (69.8) | 140 (76.9) |  |
| No | 16 (30.2) | 37 (20.3) |  |
| Unknown | 0 (0.0) | 5 (2.7) |  |
| Number of hospitalizations in the previous year | 0.50 ±0.98 | 0.46 ±1.06 | 0.726 |
| CAT total score | 22.82 ±5.56 | 21.50 ±6.00 | 0.064 |
| CCQ total score | 3.05 ±0.84 | 3.00 ±0.89 | 0.586 |
| mMRC | 3.00 [2.00, 3.00] | 3.00 [2.00, 3.00] | 0.624 |
| SGRQ total score | 58.47 ±11.95 | 57.03 ±13.18 | 0.356 |
| FEV1 % predicted | 29.39 ±9.80 | 28.97 ±9.81 | 0.724 |
| RV/TLC, % | 60.66 ±9.11 | 60.82 ±8.55 | 0.878 |
| Emphysema destruction severity, LAA -950HU | 37.38 ±6.61 | 34.82 ±9.03 | **0.016** |
| Pi10 | 2.60 ±0.29 | 2.64 ±0.30 | 0.322 |
| Chronic kidney disease | 2 (2.7) | 13 (3.9) | 0.875 |
| Congestive heart failure | 3 (4.0) | 8 (2.4) | 0.694 |
| Pulmonary Arterial Hypertension | 6 (8.0) | 25 (7.4) | 1.000 |
| Atherosclerosis | 22 (29.3) | 58 (17.2) | 0.025 |
| Myocardial Infarction | 10 (13.3) | 15 (4.5) | **0.008** |
| Coronary Artery disease | 15 (20.0) | 28 (8.3) | **0.005** |
| Cerebrovasculair accident | 5 (6.7) | 9 (2.7) | 0.169 |
| Malignancy | 10 (13.3) | 60 (17.8) | 0.446 |
| Autoimmune disorder | 5 (6.7) | 21 (6.2) | 1.000 |
| Hypertension | 30 (40.0) | 109 (32.3) | 0.257 |
| Diabetes Mellitus | 4 (5.3) | 17 (5.0) | 1.000 |
| CRP, mg/L | 2.58 ±2.89 | 3.43 ±3.80 | 0.051 |
| Data are presented as n, n (%), mean±SD or median [interquartile range], unless otherwise specified. Univariate differences were tested with Chi-square tests, independent T-tests and Mann-whitney U-tests as appropriate. BMI: Body Mass Index; ICS: inhaled corticosteroids; LABA: long acting β2 agonist; CAT: COPD assessment test; CCQ: Clinical COPD questionnaire; CCQ: Clinical COPD Questionnaire; mMRC: Modified Medical Research Council Dyspnea Scale; SGRQ: St. George’s Respiratory Questionnaire; FEV_1_: Forced Expiratory Volume in 1 second; RV/TLC: Residual Volume/Total Lung Capacity; LAA: low attenuation areas < −950 hounsfield units (HU) on the inspiratory CT scan; Pi10: 10-mm internal luminal perimeter; CRP: C-Reactive Protein. | | | |

## Supplemenentary Table S3.19: Baseline characteristics by Fluorescent light distribution width of the monocyte population (MO-Y)

|  | **Above reference interval** | **Within normal reference interval** | **p** |
| --- | --- | --- | --- |
| n | 103 | 387 |  |
| Age, years | 62.31 ±7.42 | 61.57 ±7.43 | 0.370 |
| Sex, female | 69 (67.0) | 272 (70.3) | 0.599 |
| BMI, kg/m2 | 24.39 ±4.31 | 24.25 ±4.23 | 0.766 |
| Smoking status |  |  | 0.663 |
| Current smoker | 1 (1.0) | 8 (2.1) |  |
| Ex-smoker | 102 (99.0) | 376 (97.7) |  |
| Never smoker | 0 (0.0) | 1 (0.3) |  |
| Packyears | 42.81 ±19.76 | 40.37 ±19.48 | 0.266 |
| Use of ICS or ICS/LABA | 85 (98.8) | 330 (93.8) | 0.104 |
| Number of exacerbations in the previous year | 2.00 [1.00, 3.00] | 1.00 [1.00, 3.00] | 0.189 |
| Hospitalization due to an exacerbation in the previous year |  |  | 0.704 |
| Yes | 47 (77.0) | 141 (76.6) |  |
| No | 12 (19.7) | 40 (21.7) |  |
| Unknown | 2 (3.3) | 3 (1.6) |  |
| Number of hospitalizations in the previous year | 0.40 ±0.73 | 0.50 ±1.12 | 0.433 |
| CAT total score | 21.66 ±5.66 | 21.87 ±6.02 | 0.750 |
| CCQ total score | 3.07 ±0.81 | 3.01 ±0.91 | 0.528 |
| mMRC | 3.00 [2.00, 3.00] | 3.00 [2.00, 3.00] | 0.413 |
| SGRQ total score | 57.95 ±12.22 | 57.09 ±13.28 | 0.561 |
| FEV1 % predicted | 28.49 ±9.27 | 29.18 ±9.92 | 0.521 |
| RV/TLC, % | 61.72 ±8.37 | 60.57 ±8.71 | 0.230 |
| Emphysema destruction severity, LAA -950HU | 35.35 ±8.99 | 34.99 ±8.74 | 0.721 |
| Pi10 | 2.65 ±0.28 | 2.63 ±0.30 | 0.485 |
| Chronic kidney disease | 5 (5.8) | 11 (3.2) | 0.417 |
| Congestive heart failure | 1 (1.2) | 10 (2.9) | 0.586 |
| Pulmonary Arterial Hypertension | 12 (14.0) | 20 (5.9) | **0.021** |
| Atherosclerosis | 23 (26.7) | 65 (19.1) | 0.154 |
| Myocardial Infarction | 4 (4.7) | 22 (6.5) | 0.710 |
| Coronary Artery disease | 9 (10.5) | 38 (11.1) | 1.000 |
| Cerebrovasculair accident | 5 (5.8) | 9 (2.6) | 0.255 |
| Malignancy | 15 (17.4) | 60 (17.6) | 1.000 |
| Autoimmune disorder | 8 (9.3) | 19 (5.6) | 0.307 |
| Hypertension | 29 (33.7) | 116 (34.0) | 1.000 |
| Diabetes Mellitus | 6 (7.0) | 16 (4.7) | 0.560 |
| CRP, mg/L | 3.46 ±3.55 | 3.27 ±3.63 | 0.630 |
| Data are presented as n, n (%), mean±SD or median [interquartile range], unless otherwise specified. Univariate differences were tested with Chi-square tests, independent T-tests and Mann-whitney U-tests as appropriate. BMI: Body Mass Index; ICS: inhaled corticosteroids; LABA: long acting β2 agonist; CAT: COPD assessment test; CCQ: Clinical COPD questionnaire; CCQ: Clinical COPD Questionnaire; mMRC: Modified Medical Research Council Dyspnea Scale; SGRQ: St. George’s Respiratory Questionnaire; FEV_1_: Forced Expiratory Volume in 1 second; RV/TLC: Residual Volume/Total Lung Capacity; LAA: low attenuation areas < −950 hounsfield units (HU) on the inspiratory CT scan; Pi10: 10-mm internal luminal perimeter; CRP: C-Reactive Protein. | | | |

## Supplementary Table S3.20: Baseline characteristics by Neutrophils (absolute count) (NEUT)

|  | **Above reference interval** | **Within normal reference interval** | **p** |
| --- | --- | --- | --- |
| n | 187 | 308 |  |
| Age, years | 61.74 ±7.53 | 61.65 ±7.37 | 0.895 |
| Sex, female | 129 (69.0) | 216 (70.1) | 0.867 |
| BMI, kg/m2 | 24.59 ±4.52 | 24.10 ±4.04 | 0.217 |
| Smoking status |  |  | 0.110 |
| Current smoker | 1 (0.5) | 8 (2.6) |  |
| Ex-smoker | 185 (98.9) | 298 (97.4) |  |
| Never smoker | 1 (0.5) | 0 (0.0) |  |
| Packyears | 42.24 ±21.28 | 39.96 ±18.24 | 0.210 |
| Use of ICS or ICS/LABA | 162 (95.3) | 255 (94.4) | 0.865 |
| Number of exacerbations in the previous year | 2.00 [1.00, 3.00] | 1.00 [1.00, 3.00] | 0.255 |
| Hospitalization due to an exacerbation in the previous year |  |  | 0.144 |
| Yes | 78 (83.0) | 111 (72.5) |  |
| No | 14 (14.9) | 39 (25.5) |  |
| Unknown | 2 (2.1) | 3 (2.0) |  |
| Number of hospitalizations in the previous year | 0.47 ±0.89 | 0.47 ±1.13 | 0.972 |
| CAT total score | 22.09 ±6.30 | 21.65 ±5.77 | 0.434 |
| CCQ total score | 3.10 ±0.91 | 2.97 ±0.88 | 0.103 |
| mMRC | 3.00 [2.00, 3.00] | 3.00 [2.00, 3.00] | 0.012 |
| SGRQ total score | 57.90 ±13.34 | 56.84 ±12.96 | 0.390 |
| FEV1 % predicted | 27.34 ±8.30 | 30.02 ±10.43 | **0.003** |
| RV/TLC, % | 61.74 ±8.57 | 60.27 ±8.63 | 0.067 |
| Emphysema destruction severity, LAA -950HU | 35.25 ±8.16 | 34.99 ±9.11 | 0.752 |
| Pi10 | 2.64 ±0.28 | 2.63 ±0.31 | 0.645 |
| Chronic kidney disease | 8 (4.8) | 8 (3.0) | 0.479 |
| Congestive heart failure | 4 (2.4) | 7 (2.6) | 1.000 |
| Pulmonary Arterial Hypertension | 12 (7.2) | 20 (7.5) | 1.000 |
| Atherosclerosis | 32 (19.3) | 56 (21.1) | 0.747 |
| Myocardial Infarction | 15 (9.0) | 11 (4.1) | 0.061 |
| Coronary Artery disease | 20 (12.0) | 27 (10.2) | 0.647 |
| Cerebrovasculair accident | 3 (1.8) | 11 (4.1) | 0.294 |
| Malignancy | 26 (15.7) | 49 (18.4) | 0.545 |
| Autoimmune disorder | 10 (6.0) | 18 (6.8) | 0.917 |
| Hypertension | 58 (34.9) | 89 (33.5) | 0.832 |
| Diabetes Mellitus | 10 (6.0) | 12 (4.5) | 0.638 |
| CRP, mg/L | 4.12 ±4.10 | 2.84 ±3.29 | **<0.001** |
| Data are presented as n, n (%), mean±SD or median [interquartile range], unless otherwise specified. Univariate differences were tested with Chi-square tests, independent T-tests and Mann-whitney U-tests as appropriate. BMI: Body Mass Index; ICS: inhaled corticosteroids; LABA: long acting β2 agonist; CAT: COPD assessment test; CCQ: Clinical COPD questionnaire; CCQ: Clinical COPD Questionnaire; mMRC: Modified Medical Research Council Dyspnea Scale; SGRQ: St. George’s Respiratory Questionnaire; FEV_1_: Forced Expiratory Volume in 1 second; RV/TLC: Residual Volume/Total Lung Capacity; LAA: low attenuation areas < −950 hounsfield units (HU) on the inspiratory CT scan; Pi10: 10-mm internal luminal perimeter; CRP: C-Reactive Protein. | | | |

## Supplementary Table S3.21: Baseline characteristics by Mean forward scattered light intensity of the neutrophil population (NE-FSC)

|  | **Below reference interval** | **Within normal reference interval** | **p** |
| --- | --- | --- | --- |
| n | 96 | 394 |  |
| Age, years | 61.29 ±8.24 | 61.68 ±7.20 | 0.645 |
| Sex, female | 61 (63.5) | 281 (71.3) | 0.172 |
| BMI, kg/m2 | 24.77 ±4.59 | 24.17 ±4.14 | 0.211 |
| Smoking status |  |  | 0.714 |
| Current smoker | 1 (1.0) | 8 (2.0) |  |
| Ex-smoker | 95 (99.0) | 383 (97.7) |  |
| Never smoker | 0 (0.0) | 1 (0.3) |  |
| Packyears | 41.15 (15.04) | 40.39 (19.60) | 0.723 |
| Use of ICS or ICS/LABA | 82 (91.1) | 330 (95.7) | 0.147 |
| Number of exacerbations in the previous year | 2.00 [1.00, 3.00] | 2.00 [1.00, 3.00] | 0.489 |
| Hospitalization due to an exacerbation in the previous year |  |  | 0.077 |
| Yes | 49 (87.5) | 139 (73.2) |  |
| No | 6 (10.7) | 47 (24.7) |  |
| Unknown | 1 (1.8) | 4 (2.1) |  |
| Number of hospitalizations in the previous year | 0.64 ±1.34 | 0.43 ±0.96 | 0.087 |
| CAT total score | 21.46 ±6.51 | 21.94 ±5.86 | 0.485 |
| CCQ total score | 3.02 ±0.97 | 3.02 ±0.88 | 0.979 |
| mMRC | 3.00 [2.00, 3.00] | 3.00 [2.00, 3.00] | 0.766 |
| SGRQ total score | 57.09 ±13.49 | 57.41 ±13.00 | 0.833 |
| FEV1 % predicted | 27.58 ±8.87 | 29.35 ±9.95 | 0.111 |
| RV/TLC, % | 60.38 ±7.67 | 60.91 ±8.88 | 0.590 |
| Emphysema destruction severity, LAA -950HU | 33.94 ±8.79 | 35.27 ±8.72 | 0.196 |
| Pi10 | 2.68 ±0.35 | 2.62 ±0.29 | 0.076 |
| Chronic kidney disease | 2 (2.4) | 14 (4.1) | 0.711 |
| Congestive heart failure | 3 (3.7) | 8 (2.3) | 0.764 |
| Pulmonary Arterial Hypertension | 4 (4.9) | 27 (7.8) | 0.491 |
| Atherosclerosis | 16 (19.5) | 70 (20.3) | 0.996 |
| Myocardial Infarction | 6 (7.3) | 20 (5.8) | 0.794 |
| Coronary Artery disease | 8 (9.8) | 37 (10.7) | 0.955 |
| Cerebrovasculair accident | 6 (7.3) | 8 (2.3) | 0.052 |
| Malignancy | 16 (19.5) | 59 (17.1) | 0.723 |
| Autoimmune disorder | 6 (7.3) | 21 (6.1) | 0.874 |
| Hypertension | 26 (31.7) | 117 (33.9) | 0.802 |
| Diabetes Mellitus | 4 (4.9) | 18 (5.2) | 1.000 |
| CRP, mg/L | 4.19 ±4.36 | 3.14 ±3.46 | **0.012** |
| Data are presented as n, n (%), mean±SD or median [interquartile range], unless otherwise specified. Univariate differences were tested with Chi-square tests, independent T-tests and Mann-whitney U-tests as appropriate. BMI: Body Mass Index; ICS: inhaled corticosteroids; LABA: long acting β2 agonist; CAT: COPD assessment test; CCQ: Clinical COPD questionnaire; CCQ: Clinical COPD Questionnaire; mMRC: Modified Medical Research Council Dyspnea Scale; SGRQ: St. George’s Respiratory Questionnaire; FEV_1_: Forced Expiratory Volume in 1 second; RV/TLC: Residual Volume/Total Lung Capacity; LAA: low attenuation areas < −950 hounsfield units (HU) on the inspiratory CT scan; Pi10: 10-mm internal luminal perimeter; CRP: C-Reactive Protein. | | | |

## Supplementary Table S3.22: Baseline characteristics by Neutrophil reactivity intensity (Neutrophil reactivity intensity)

|  | **Above reference interval** | **Within normal reference interval** | **p** |
| --- | --- | --- | --- |
| n | 39 | 437 |  |
| Age, years | 62.03 ±7.83 | 61.64 ±7.41 | 0.758 |
| Sex, female | 30 (76.9) | 303 (69.3) | 0.419 |
| BMI, kg/m2 | 23.74 ±4.87 | 24.30 ±4.14 | 0.426 |
| Smoking status |  |  | 0.910 |
| Current smoker | 1 (2.6) | 8 (1.8) |  |
| Ex-smoker | 38 (97.4) | 426 (97.9) |  |
| Never smoker | 0 (0.0) | 1 (0.2) |  |
| Packyears | 44.71 ±17.68 | 40.63 ±19.68 | 0.217 |
| Use of ICS or ICS/LABA | 31 (100.0) | 371 (94.6) | 0.372 |
| Number of exacerbations in the previous year | 1.00 [1.00, 2.50] | 2.00 [1.00, 3.00] | 0.898 |
| Hospitalization due to an exacerbation in the previous year |  |  | 0.204 |
| Yes | 17 (65.4) | 163 (77.3) |  |
| No | 9 (34.6) | 43 (20.4) |  |
| Unknown | 0 (0.0) | 5 (2.4) |  |
| Number of hospitalizations in the previous year | 0.62 ±1.11 | 0.45 ±1.03 | 0.334 |
| CAT total score | 22.74 ±6.06 | 21.71 ±5.96 | 0.310 |
| CCQ total score | 3.11 ±0.94 | 3.00 ±0.89 | 0.496 |
| mMRC | 3.00 [2.00, 3.00] | 3.00 [2.00, 3.00] | 0.599 |
| SGRQ total score | 57.93 ±13.22 | 57.21 ±13.12 | 0.746 |
| FEV1 % predicted | 28.85 ±9.54 | 29.22 ±9.85 | 0.825 |
| RV/TLC, % | 61.94 ±9.82 | 60.60 ±8.55 | 0.356 |
| Emphysema destruction severity, LAA -950HU | 38.20 ±8.34 | 34.81 ±8.83 | **0.029** |
| Pi10 | 2.59 ±0.26 | 2.64 ±0.30 | 0.385 |
| Chronic kidney disease | 1 (3.1) | 15 (3.9) | 1.000 |
| Congestive heart failure | 0 (0.0) | 10 (2.6) | 0.748 |
| Pulmonary Arterial Hypertension | 5 (15.6) | 26 (6.8) | 0.137 |
| Atherosclerosis | 6 (18.8) | 78 (20.3) | 1.000 |
| Myocardial Infarction | 0 (0.0) | 24 (6.2) | 0.289 |
| Coronary Artery disease | 2 (6.2) | 43 (11.2) | 0.572 |
| Cerebrovasculair accident | 1 (3.1) | 12 (3.1) | 1.000 |
| Malignancy | 4 (12.5) | 67 (17.4) | 0.642 |
| Autoimmune disorder | 4 (12.5) | 22 (5.7) | 0.252 |
| Hypertension | 7 (21.9) | 137 (35.6) | 0.170 |
| Diabetes Mellitus | 2 (6.2) | 20 (5.2) | 1.000 |
| CRP, mg/L | 3.47 ±3.79 | 3.33 ±3.68 | 0.817 |
| Data are presented as n, n (%), mean±SD or median [interquartile range], unless otherwise specified. Univariate differences were tested with Chi-square tests, independent T-tests and Mann-whitney U-tests as appropriate. BMI: Body Mass Index; ICS: inhaled corticosteroids; LABA: long acting β2 agonist; CAT: COPD assessment test; CCQ: Clinical COPD questionnaire; CCQ: Clinical COPD Questionnaire; mMRC: Modified Medical Research Council Dyspnea Scale; SGRQ: St. George’s Respiratory Questionnaire; FEV_1_: Forced Expiratory Volume in 1 second; RV/TLC: Residual Volume/Total Lung Capacity; LAA: low attenuation areas < −950 hounsfield units (HU) on the inspiratory CT scan; Pi10: 10-mm internal luminal perimeter; CRP: C-Reactive Protein. | | | |

## Supplementary Table S3.23: Baseline characteristics by Neutrophil granularity intensity (NEUT-GI)

|  | **Above reference interval** | **Within normal reference interval** | **p** |
| --- | --- | --- | --- |
| n | 40 | 440 |  |
| Age, years | 62.77 ±8.20 | 61.50 ±7.36 | 0.298 |
| Sex, female | 23 (57.5) | 310 (70.5) | 0.128 |
| BMI, kg/m2 | 24.77 ±4.48 | 24.23 ±4.22 | 0.447 |
| Smoking status |  |  | 0.665 |
| Current smoker | 0 (0.0) | 8 (1.8) |  |
| Ex-smoker | 39 (100.0) | 430 (97.9) |  |
| Never smoker | 0 (0.0) | 1 (0.2) |  |
| Packyears | 44.73 ±24.79 | 40.50 ±19.19 | 0.194 |
| Use of ICS or ICS/LABA | 30 (85.7) | 372 (95.4) | **0.042** |
| Number of exacerbations in the previous year | 1.50 [1.00, 2.25] | 2.00 [1.00, 3.00] | 0.882 |
| Hospitalization due to an exacerbation in the previous year |  |  | 0.603 |
| Yes | 13 (86.7) | 168 (76.0) |  |
| No | 2 (13.3) | 48 (21.7) |  |
| Unknown | 0 (0.0) | 5 (2.3) |  |
| Number of hospitalizations in the previous year | 0.38 ±0.67 | 0.47 ±0.96 | 0.545 |
| CAT total score | 22.33 ±6.14 | 21.78 ±6.00 | 0.593 |
| CCQ total score | 3.16 ±1.01 | 3.00 ±0.88 | 0.300 |
| mMRC | 3.00 [2.00, 3.00] | 3.00 [2.00, 3.00] | 0.503 |
| SGRQ total score | 58.40 ±14.37 | 57.15 ±13.06 | 0.578 |
| FEV1 % predicted | 30.22 ±10.07 | 28.75 ±9.70 | 0.358 |
| RV/TLC, % | 60.47 ±9.35 | 60.85 ±8.54 | 0.791 |
| Emphysema destruction severity, LAA -950HU | 34.05 ±8.33 | 35.12 ±8.83 | 0.462 |
| Pi10 | 2.66 ±0.33 | 2.63 ±0.30 | 0.652 |
| Chronic kidney disease | 1 (2.7) | 15 (3.9) | 1.000 |
| Congestive heart failure | 1 (2.7) | 10 (2.6) | 1.000 |
| Pulmonary Arterial Hypertension | 3 (8.1) | 28 (7.3) | 1.000 |
| Atherosclerosis | 8 (21.6) | 75 (19.6) | 0.935 |
| Myocardial Infarction | 4 (10.8) | 22 (5.7) | 0.388 |
| Coronary Artery disease | 6 (16.2) | 40 (10.4) | 0.425 |
| Cerebrovasculair accident | 0 (0.0) | 13 (3.4) | 0.521 |
| Malignancy | 5 (13.5) | 67 (17.5) | 0.700 |
| Autoimmune disorder | 3 (8.1) | 25 (6.5) | 0.982 |
| Hypertension | 12 (32.4) | 130 (33.9) | 0.997 |
| Diabetes Mellitus | 1 (2.7) | 20 (5.2) | 0.782 |
| CRP, mg/L | 3.91 ±4.09 | 3.32 ±3.66 | 0.334 |
| Data are presented as n, n (%), mean±SD or median [interquartile range], unless otherwise specified. Univariate differences were tested with Chi-square tests, independent T-tests and Mann-whitney U-tests as appropriate. BMI: Body Mass Index; ICS: inhaled corticosteroids; LABA: long acting β2 agonist; CAT: COPD assessment test; CCQ: Clinical COPD questionnaire; CCQ: Clinical COPD Questionnaire; mMRC: Modified Medical Research Council Dyspnea Scale; SGRQ: St. George’s Respiratory Questionnaire; FEV_1_: Forced Expiratory Volume in 1 second; RV/TLC: Residual Volume/Total Lung Capacity; LAA: low attenuation areas < −950 hounsfield units (HU) on the inspiratory CT scan; Pi10: 10-mm internal luminal perimeter; CRP: C-Reactive Protein. | | | |

## Supplementary Table S3.24: Baseline characteristics by Platelet large cell ratio (P-LCR)

|  | **Below reference interval** | **Within normal reference interval** | **p** |
| --- | --- | --- | --- |
| n | 111 | 386 |  |
| Age, years | 60.73 ±7.26 | 61.97 ±7.43 | 0.120 |
| Sex, female | 82 (73.9) | 263 (68.1) | 0.299 |
| BMI, kg/m2 | 23.48 ±4.08 | 24.50 ±4.23 | **0.024** |
| Smoking status |  |  | 0.623 |
| Current smoker | 1 (0.9) | 8 (2.1) |  |
| Ex-smoker | 109 (99.1) | 376 (97.7) |  |
| Never smoker | 0 (0.0) | 1 (0.3) |  |
| Packyears | 39.87 ±17.87 | 41.15 ±19.96 | 0.544 |
| Use of ICS or ICS/LABA | 96 (95.0) | 324 (95.0) | 1.000 |
| Number of exacerbations in the previous year | 2.00 [1.00, 3.00] | 2.00 [1.00, 3.00] | 0.551 |
| Hospitalization due to an exacerbation in the previous year |  |  | 0.451 |
| Yes | 47 (82.5) | 142 (74.3) |  |
| No | 9 (15.8) | 44 (23.0) |  |
| Unknown | 1 (1.8) | 5 (2.6) |  |
| Number of hospitalizations in the previous year | 0.42 ±0.79 | 0.48 ±1.11 | 0.590 |
| CAT total score | 21.80 ±6.10 | 21.87 ±5.98 | 0.914 |
| CCQ total score | 3.06 ±0.82 | 3.01 ±0.92 | 0.627 |
| mMRC | 3.00 [2.00, 3.00] | 3.00 [2.00, 3.00] | 0.326 |
| SGRQ total score | 58.42 ±13.20 | 57.03 ±13.08 | 0.336 |
| FEV1 % predicted | 28.20 ±7.90 | 29.22 ±10.22 | 0.333 |
| RV/TLC, % | 60.92 ±8.55 | 60.84 ±8.64 | 0.939 |
| Emphysema destruction severity, LAA -950HU | 35.11 ±9.48 | 35.09 ±8.50 | 0.986 |
| Pi10 | 2.65 ±0.31 | 2.63 ±0.30 | 0.467 |
| Chronic kidney disease | 6 (6.2) | 9 (2.7) | 0.176 |
| Congestive heart failure | 2 (2.1) | 9 (2.7) | 1.000 |
| Pulmonary Arterial Hypertension | 9 (9.3) | 23 (6.8) | 0.552 |
| Atherosclerosis | 17 (17.5) | 70 (20.8) | 0.576 |
| Myocardial Infarction | 6 (6.2) | 19 (5.6) | 1.000 |
| Coronary Artery disease | 10 (10.3) | 36 (10.7) | 1.000 |
| Cerebrovasculair accident | 2 (2.1) | 12 (3.6) | 0.682 |
| Malignancy | 12 (12.4) | 63 (18.7) | 0.194 |
| Autoimmune disorder | 8 (8.2) | 20 (5.9) | 0.560 |
| Hypertension | 33 (34.0) | 115 (34.1) | 1.000 |
| Diabetes Mellitus | 3 (3.1) | 19 (5.6) | 0.457 |
| CRP, mg/L | 3.85 ±4.24 | 3.16 ±3.45 | 0.077 |
| Data are presented as n, n (%), mean±SD or median [interquartile range], unless otherwise specified. Univariate differences were tested with Chi-square tests, independent T-tests and Mann-whitney U-tests as appropriate. BMI: Body Mass Index; ICS: inhaled corticosteroids; LABA: long acting β2 agonist; CAT: COPD assessment test; CCQ: Clinical COPD questionnaire; CCQ: Clinical COPD Questionnaire; mMRC: Modified Medical Research Council Dyspnea Scale; SGRQ: St. George’s Respiratory Questionnaire; FEV_1_: Forced Expiratory Volume in 1 second; RV/TLC: Residual Volume/Total Lung Capacity; LAA: low attenuation areas < −950 hounsfield units (HU) on the inspiratory CT scan; Pi10: 10-mm internal luminal perimeter; CRP: C-Reactive Protein. | | | |

## Supplementary Table S3.25: Baseline characteristics by Platelet count (PLT)

|  | **Above reference interval** | **Within normal reference interval** | **p** |
| --- | --- | --- | --- |
| n | 50 | 440 |  |
| Age, years | 59.28 ±6.87 | 61.89 ±7.44 | **0.018** |
| Sex, female | 40 (80.0) | 299 (68.0) | 0.113 |
| BMI, kg/m2 | 23.81 ±4.33 | 24.33 ±4.23 | 0.407 |
| Smoking status |  |  | 0.599 |
| Current smoker | 0 (0.0) | 8 (1.8) |  |
| Ex-smoker | 49 (100.0) | 430 (97.9) |  |
| Never smoker | 0 (0.0) | 1 (0.2) |  |
| Packyears | 42.54 ±21.62 | 40.62 ±19.33 | 0.512 |
| Use of ICS or ICS/LABA | 40 (93.0) | 374 (94.9) | 0.865 |
| Number of exacerbations in the previous year | 2.00 [1.00, 3.00] | 2.00 [1.00, 3.00] | 0.070 |
| Hospitalization due to an exacerbation in the previous year |  |  | 0.659 |
| Yes | 21 (80.8) | 166 (75.5) |  |
| No | 4 (15.4) | 49 (22.3) |  |
| Unknown | 1 (3.8) | 5 (2.3) |  |
| Number of hospitalizations in the previous year | 0.38 ±0.64 | 0.48 ±1.08 | 0.508 |
| CAT total score | 21.47 ±6.91 | 21.86 ±5.89 | 0.666 |
| CCQ total score | 2.94 ±0.93 | 3.03 ±0.89 | 0.502 |
| mMRC | 2.50 [2.00, 3.00] | 3.00 [2.00, 3.00] | 0.343 |
| SGRQ total score | 55.92 ±14.13 | 57.44 ±12.96 | 0.442 |
| FEV1 % predicted | 27.62 ±8.24 | 29.19 ±9.94 | 0.280 |
| RV/TLC, % | 61.89 ±8.69 | 60.58 ±8.58 | 0.306 |
| Emphysema destruction severity, LAA -950HU | 35.43 ±8.91 | 35.04 ±8.71 | 0.767 |
| Pi10 | 2.67 ±0.33 | 2.63 ±0.30 | 0.403 |
| Chronic kidney disease | 3 (6.7) | 12 (3.1) | 0.431 |
| Congestive heart failure | 1 (2.2) | 10 (2.6) | 1.000 |
| Pulmonary Arterial Hypertension | 2 (4.4) | 30 (7.9) | 0.602 |
| Atherosclerosis | 7 (15.6) | 78 (20.4) | 0.565 |
| Myocardial Infarction | 4 (8.9) | 22 (5.8) | 0.616 |
| Coronary Artery disease | 6 (13.3) | 40 (10.5) | 0.740 |
| Cerebrovasculair accident | 0 (0.0) | 12 (3.1) | 0.466 |
| Malignancy | 5 (11.1) | 66 (17.3) | 0.401 |
| Autoimmune disorder | 4 (8.9) | 23 (6.0) | 0.672 |
| Hypertension | 14 (31.1) | 131 (34.3) | 0.795 |
| Diabetes Mellitus | 3 (6.7) | 18 (4.7) | 0.834 |
| CRP, mg/L | 3.80 ±4.59 | 3.27 ±3.54 | 0.335 |
| Data are presented as n, n (%), mean±SD or median [interquartile range], unless otherwise specified. Univariate differences were tested with Chi-square tests, independent T-tests and Mann-whitney U-tests as appropriate. BMI: Body Mass Index; ICS: inhaled corticosteroids; LABA: long acting β2 agonist; CAT: COPD assessment test; CCQ: Clinical COPD questionnaire; CCQ: Clinical COPD Questionnaire; mMRC: Modified Medical Research Council Dyspnea Scale; SGRQ: St. George’s Respiratory Questionnaire; FEV_1_: Forced Expiratory Volume in 1 second; RV/TLC: Residual Volume/Total Lung Capacity; LAA: low attenuation areas < −950 hounsfield units (HU) on the inspiratory CT scan; Pi10: 10-mm internal luminal perimeter; CRP: C-Reactive Protein. | | | |

## Supplementary Table S3.26: Baseline characteristics by Reactive lymphocytes (RE-LYMP)

|  | **Above reference interval** | **Within normal reference interval** | **p** |
| --- | --- | --- | --- |
| n | 110 | 367 |  |
| Age, years | 59.80 ±6.79 | 62.16 ±7.48 | **0.003** |
| Sex, female | 91 (82.7) | 240 (65.4) | **0.001** |
| BMI, kg/m2 | 25.15 ±4.83 | 23.93 ±3.98 | **0.008** |
| Smoking status |  |  | 0.858 |
| Current smoker | 2 (1.8) | 7 (1.9) |  |
| Ex-smoker | 108 (98.2) | 357 (97.8) |  |
| Never smoker | 0 (0.0) | 1 (0.3) |  |
| Packyears | 42.80 ±19.44 | 40.37 ±19.74 | 0.260 |
| Use of ICS or ICS/LABA | 90 (97.8) | 312 (94.3) | 0.262 |
| Number of exacerbations in the previous year | 2.00 [1.00, 3.00] | 2.00 [1.00, 3.00] | **0.012** |
| Hospitalization due to an exacerbation in the previous year |  |  | 0.290 |
| Yes | 50 (83.3) | 129 (73.3) |  |
| No | 9 (15.0) | 43 (24.4) |  |
| Unknown | 1 (1.7) | 4 (2.3) |  |
| Number of hospitalizations in the previous year | 0.54 ±1.08 | 0.44 ±1.04 | 0.380 |
| CAT total score | 21.51 ±5.49 | 21.88 ±6.11 | 0.581 |
| CCQ total score | 2.97 ±0.83 | 3.04 ±0.91 | 0.503 |
| mMRC | 2.00 [2.00, 3.00] | 3.00 [2.00, 3.00] | 0.030 |
| SGRQ total score | 57.27 ±12.00 | 57.14 ±13.36 | 0.927 |
| FEV1 % predicted | 29.93 ±9.29 | 28.68 ±9.89 | 0.240 |
| RV/TLC, % | 59.86 ±7.55 | 61.15 ±8.91 | 0.169 |
| Emphysema destruction severity, LAA -950HU | 34.25 ±9.92 | 35.40 ±8.42 | 0.243 |
| Pi10 | 2.66 ±0.30 | 2.63 ±0.30 | 0.374 |
| Chronic kidney disease | 2 (2.1) | 14 (4.3) | 0.479 |
| Congestive heart failure | 2 (2.1) | 9 (2.8) | 0.988 |
| Pulmonary Arterial Hypertension | 8 (8.3) | 23 (7.1) | 0.860 |
| Atherosclerosis | 18 (18.8) | 66 (20.4) | 0.829 |
| Myocardial Infarction | 7 (7.3) | 18 (5.6) | 0.705 |
| Coronary Artery disease | 10 (10.4) | 36 (11.1) | 0.988 |
| Cerebrovasculair accident | 3 (3.1) | 10 (3.1) | 1.000 |
| Malignancy | 14 (14.6) | 58 (18.0) | 0.538 |
| Autoimmune disorder | 9 (9.4) | 17 (5.3) | 0.220 |
| Hypertension | 28 (29.2) | 113 (35.0) | 0.349 |
| Diabetes Mellitus | 8 (8.3) | 13 (4.0) | 0.152 |
| CRP, mg/L | 4.17 ±3.87 | 3.09 ±3.50 | **0.006** |
| Data are presented as n, n (%), mean±SD or median [interquartile range], unless otherwise specified. Univariate differences were tested with Chi-square tests, independent T-tests and Mann-whitney U-tests as appropriate. BMI: Body Mass Index; ICS: inhaled corticosteroids; LABA: long acting β2 agonist; CAT: COPD assessment test; CCQ: Clinical COPD questionnaire; CCQ: Clinical COPD Questionnaire; mMRC: Modified Medical Research Council Dyspnea Scale; SGRQ: St. George’s Respiratory Questionnaire; FEV_1_: Forced Expiratory Volume in 1 second; RV/TLC: Residual Volume/Total Lung Capacity; LAA: low attenuation areas < −950 hounsfield units (HU) on the inspiratory CT scan; Pi10: 10-mm internal luminal perimeter; CRP: C-Reactive Protein. | | | |

| Supplementary Table S4a: Pearson correlation coefficients and p-values between continuous clinical variables and cell population data parameters | | | | | | | | | | | | |
| --- | --- | --- | --- | --- | --- | --- | --- | --- | --- | --- | --- | --- |
|  | **AS-LYMP** | **EO** | **EO-X** | **EO-Y** | **EO-Z** | **HGB** | **IG** | **LYMPH** | **LY-X** | **LY-Y** | **LY-Z** | **MacroR** |
| **CRP** | 0.1 (p = 0.032) | 0.11 (p = 0.013) | 0.05 (p = 0.301) | -0.01 (p = 0.805) | 0.03 (p = 0.471) | -0.09 (p = 0.034) | 0.09 (p = 0.036) | -0.16 (p < 0.001) | 0.05 (p = 0.234) | 0.01 (p = 0.758) | -0.02 (p = 0.61) | -0.04 (p = 0.338) |
| **Pi10** | -0.02 (p = 0.719) | 0.07 (p = 0.136) | 0.01 (p = 0.769) | 0.07 (p = 0.116) | -0.01 (p = 0.768) | -0.03 (p = 0.488) | 0.04 (p = 0.393) | -0.05 (p = 0.282) | 0.06 (p = 0.178) | 0.02 (p = 0.638) | -0.01 (p = 0.872) | -0.05 (p = 0.302) |
| **VI 950%** | -0.04 (p = 0.349) | -0.05 (p = 0.294) | -0.06 (p = 0.159) | -0.01 (p = 0.814) | -0.06 (p = 0.203) | 0.2 (p < 0.001) | -0.03 (p = 0.586) | -0.04 (p = 0.363) | -0.13 (p = 0.005) | -0.02 (p = 0.735) | 0.06 (p = 0.231) | 0.01 (p = 0.781) |
| **RV/TLC** | -0.1 (p = 0.026) | 0.08 (p = 0.069) | -0.02 (p = 0.682) | -0.07 (p = 0.109) | -0.03 (p = 0.506) | -0.06 (p = 0.149) | 0 (p = 0.994) | -0.09 (p = 0.043) | -0.07 (p = 0.099) | -0.1 (p = 0.021) | -0.07 (p = 0.109) | 0.03 (p = 0.577) |
| **FEV1 %pred** | 0.11 (p = 0.014) | -0.03 (p = 0.565) | 0.03 (p = 0.536) | 0.07 (p = 0.119) | 0.03 (p = 0.482) | -0.02 (p = 0.58) | -0.03 (p = 0.439) | 0.1 (p = 0.026) | 0.03 (p = 0.446) | 0.07 (p = 0.111) | 0.1 (p = 0.028) | -0.04 (p = 0.35) |
| **CAT total score** | 0.02 (p = 0.732) | 0.04 (p = 0.379) | 0.03 (p = 0.464) | -0.02 (p = 0.596) | 0.05 (p = 0.248) | -0.09 (p = 0.046) | 0.02 (p = 0.681) | 0 (p = 0.926) | 0.03 (p = 0.489) | -0.03 (p = 0.558) | 0 (p = 0.952) | -0.01 (p = 0.895) |
| **CCQ total score** | 0.03 (p = 0.533) | 0.03 (p = 0.508) | 0 (p = 0.917) | -0.04 (p = 0.393) | 0.03 (p = 0.486) | -0.03 (p = 0.513) | 0.04 (p = 0.339) | 0.01 (p = 0.877) | 0.03 (p = 0.517) | -0.01 (p = 0.875) | -0.02 (p = 0.64) | 0.01 (p = 0.867) |
| **SGRQ total score** | 0.01 (p = 0.857) | 0.08 (p = 0.079) | 0.03 (p = 0.509) | 0.01 (p = 0.772) | 0.05 (p = 0.268) | -0.05 (p = 0.323) | 0.09 (p = 0.056) | 0 (p = 0.968) | 0.01 (p = 0.89) | 0 (p = 0.964) | -0.09 (p = 0.046) | -0.01 (p = 0.867) |
| **Packyears** | -0.01 (p = 0.764) | 0.01 (p = 0.848) | -0.13 (p = 0.005) | -0.1 (p = 0.029) | -0.11 (p = 0.013) | 0.01 (p = 0.82) | 0.15 (p = 0.001) | -0.05 (p = 0.269) | 0.03 (p = 0.461) | 0 (p = 0.996) | -0.13 (p = 0.004) | 0.02 (p = 0.738) |
| **Number of hospitalizations** | 0 (p = 0.991) | -0.05 (p = 0.453) | 0.06 (p = 0.36) | 0.06 (p = 0.346) | 0.11 (p = 0.076) | 0.17 (p = 0.009) | -0.06 (p = 0.372) | 0.01 (p = 0.87) | 0.06 (p = 0.339) | 0.03 (p = 0.63) | 0.21 (p < 0.001) | -0.02 (p = 0.77) |
| **Number of exacerbations** | 0.08 (p = 0.081) | 0.07 (p = 0.13) | -0.01 (p = 0.788) | 0.03 (p = 0.567) | 0 (p = 0.962) | -0.09 (p = 0.052) | 0.15 (p = 0.001) | 0.02 (p = 0.589) | 0.03 (p = 0.564) | 0.1 (p = 0.026) | 0.02 (p = 0.624) | -0.06 (p = 0.159) |
| **BMI** | 0.01 (p = 0.841) | 0.06 (p = 0.216) | -0.01 (p = 0.829) | 0.03 (p = 0.474) | -0.01 (p = 0.816) | -0.05 (p = 0.276) | 0.08 (p = 0.092) | 0.01 (p = 0.827) | 0.07 (p = 0.116) | 0.04 (p = 0.346) | 0.05 (p = 0.315) | 0 (p = 0.95) |
| **Age** | -0.13 (p = 0.005) | 0.01 (p = 0.834) | 0.03 (p = 0.465) | 0.06 (p = 0.17) | 0.03 (p = 0.512) | 0 (p = 0.937) | 0.06 (p = 0.155) | -0.17 (p < 0.001) | 0.06 (p = 0.184) | -0.07 (p = 0.11) | -0.07 (p = 0.13) | 0.08 (p = 0.071) |

| Supplementary Table S4b: Pearson correlation coefficients and p-values between continuous clinical variables and cell population data parameters | | | | | | | | | | | | |
| --- | --- | --- | --- | --- | --- | --- | --- | --- | --- | --- | --- | --- |
|  | **MicroR** | **MONO** | **MO-X** | **MO-Y** | **MO-Z** | **NEUT** | **NEUT-GI** | **NEUT-RI** | **NE-Z** | **P-LCR** | **PLT** | **RE-LYMP** |
| **CRP** | 0.14 (p = 0.002) | 0.15 (p = 0.001) | 0.17 (p < 0.001) | 0.01 (p = 0.848) | -0.11 (p = 0.013) | 0.2 (p < 0.001) | 0.12 (p = 0.01) | 0 (p = 0.913) | -0.1 (p = 0.023) | -0.03 (p = 0.523) | 0.08 (p = 0.093) | 0.07 (p = 0.12) |
| **Pi10** | -0.03 (p = 0.58) | 0.02 (p = 0.635) | 0.06 (p = 0.19) | 0 (p = 0.939) | -0.01 (p = 0.87) | 0.05 (p = 0.279) | 0.04 (p = 0.339) | -0.04 (p = 0.427) | -0.07 (p = 0.134) | -0.01 (p = 0.773) | 0 (p = 0.954) | 0.02 (p = 0.635) |
| **VI 950%** | -0.03 (p = 0.566) | 0 (p = 0.941) | -0.22 (p < 0.001) | 0.05 (p = 0.239) | 0.04 (p = 0.442) | -0.01 (p = 0.796) | -0.19 (p < 0.001) | 0.09 (p = 0.041) | 0.09 (p = 0.047) | 0.02 (p = 0.645) | 0.01 (p = 0.83) | -0.05 (p = 0.239) |
| **RV/TLC** | -0.02 (p = 0.594) | 0.06 (p = 0.203) | -0.01 (p = 0.807) | -0.04 (p = 0.393) | 0.07 (p = 0.115) | 0.05 (p = 0.293) | -0.04 (p = 0.364) | 0 (p = 0.943) | 0.06 (p = 0.199) | 0.05 (p = 0.224) | -0.01 (p = 0.768) | -0.08 (p = 0.077) |
| **FEV1 %pred** | 0.04 (p = 0.344) | -0.08 (p = 0.071) | 0.01 (p = 0.755) | 0.05 (p = 0.295) | 0.01 (p = 0.802) | -0.1 (p = 0.034) | 0.01 (p = 0.837) | 0.05 (p = 0.223) | 0.01 (p = 0.88) | -0.05 (p = 0.308) | -0.05 (p = 0.237) | 0.06 (p = 0.177) |
| **CAT total score** | 0.04 (p = 0.415) | -0.08 (p = 0.099) | 0.01 (p = 0.913) | -0.05 (p = 0.317) | 0.04 (p = 0.433) | 0.06 (p = 0.222) | 0.01 (p = 0.853) | 0 (p = 0.928) | 0.02 (p = 0.597) | -0.07 (p = 0.146) | 0.04 (p = 0.363) | -0.04 (p = 0.384) |
| **CCQ total score** | 0.02 (p = 0.648) | 0 (p = 0.963) | 0.01 (p = 0.869) | -0.01 (p = 0.87) | 0.02 (p = 0.734) | 0.08 (p = 0.098) | -0.01 (p = 0.876) | 0 (p = 0.993) | -0.04 (p = 0.388) | -0.07 (p = 0.125) | 0.04 (p = 0.345) | 0 (p = 0.972) |
| **SGRQ total score** | 0.04 (p = 0.376) | -0.05 (p = 0.319) | -0.02 (p = 0.7) | -0.01 (p = 0.891) | -0.03 (p = 0.517) | 0.09 (p = 0.043) | -0.03 (p = 0.493) | 0 (p = 0.998) | -0.04 (p = 0.335) | -0.06 (p = 0.206) | 0.01 (p = 0.788) | 0 (p = 0.954) |
| **Packyears** | 0.08 (p = 0.092) | 0.12 (p = 0.01) | 0 (p = 0.961) | 0.02 (p = 0.604) | -0.06 (p = 0.176) | 0.06 (p = 0.176) | 0.14 (p = 0.003) | 0 (p = 0.961) | 0.02 (p = 0.645) | 0.02 (p = 0.723) | -0.01 (p = 0.775) | 0.02 (p = 0.621) |
| **Number of hospitalizations** | -0.04 (p = 0.517) | -0.04 (p = 0.584) | -0.07 (p = 0.284) | -0.01 (p = 0.905) | 0.03 (p = 0.6) | -0.09 (p = 0.139) | -0.07 (p = 0.308) | 0.07 (p = 0.304) | 0.06 (p = 0.367) | -0.01 (p = 0.906) | -0.06 (p = 0.38) | -0.08 (p = 0.221) |
| **Number of exacerbations** | 0.02 (p = 0.652) | -0.06 (p = 0.226) | 0 (p = 0.993) | 0.01 (p = 0.746) | -0.06 (p = 0.181) | 0.13 (p = 0.004) | -0.05 (p = 0.292) | 0.09 (p = 0.055) | -0.04 (p = 0.432) | 0 (p = 0.984) | 0.06 (p = 0.16) | 0.16 (p < 0.001) |
| **BMI** | 0.08 (p = 0.093) | -0.02 (p = 0.671) | 0.13 (p = 0.005) | -0.01 (p = 0.797) | -0.07 (p = 0.106) | 0.06 (p = 0.17) | 0.1 (p = 0.021) | -0.05 (p = 0.3) | -0.09 (p = 0.058) | 0.11 (p = 0.017) | -0.06 (p = 0.219) | 0.1 (p = 0.028) |
| **Age** | 0.02 (p = 0.681) | -0.01 (p = 0.767) | 0.02 (p = 0.732) | 0.02 (p = 0.655) | -0.03 (p = 0.559) | 0.02 (p = 0.646) | 0.04 (p = 0.383) | 0.02 (p = 0.597) | 0.05 (p = 0.224) | -0.01 (p = 0.862) | -0.19 (p < 0.001) | -0.19 (p < 0.001) |


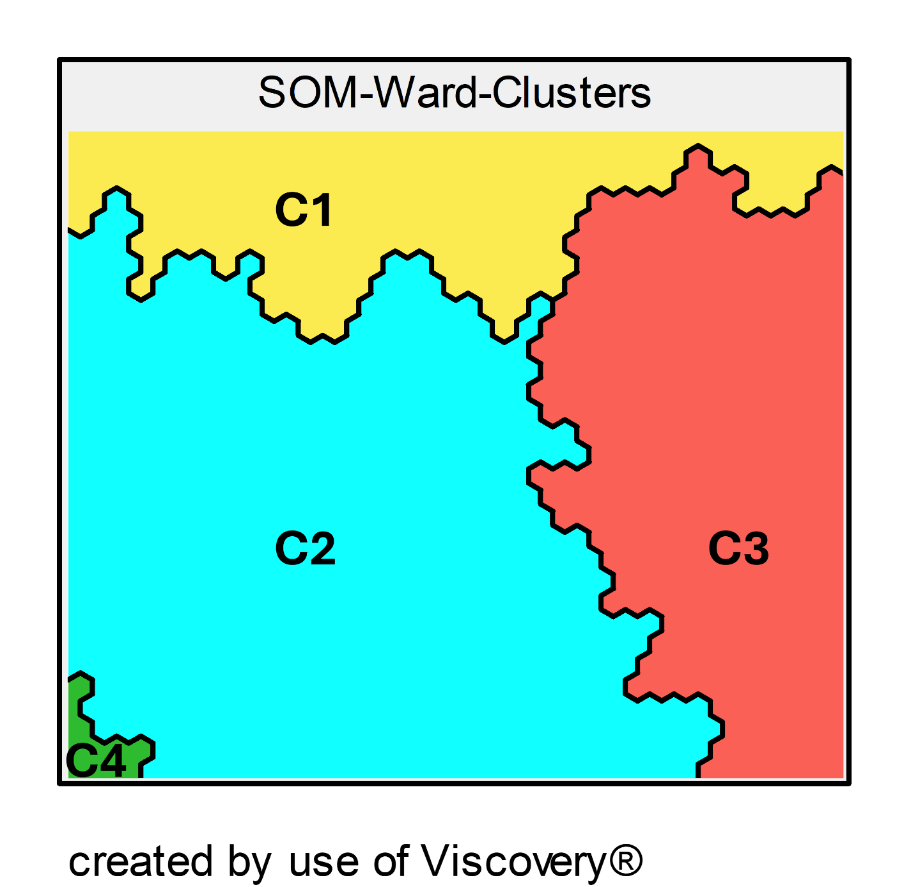


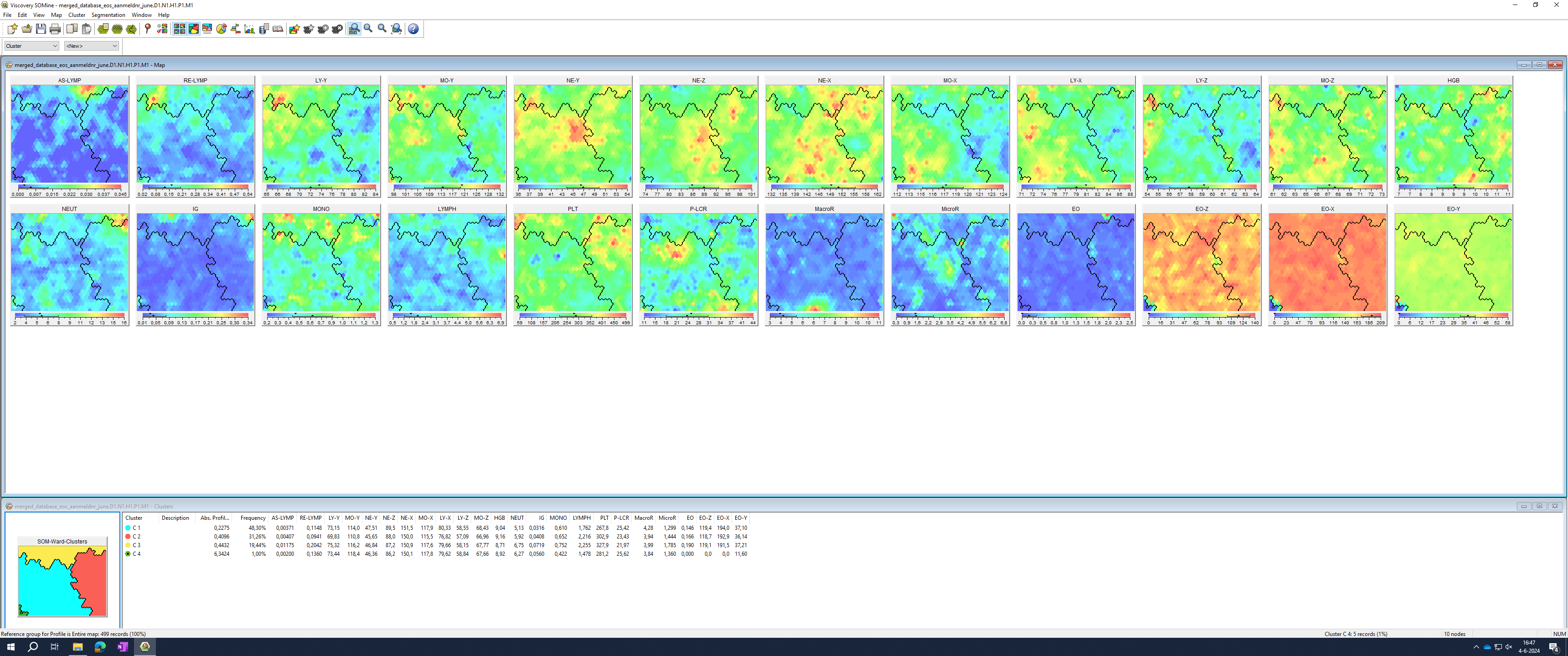

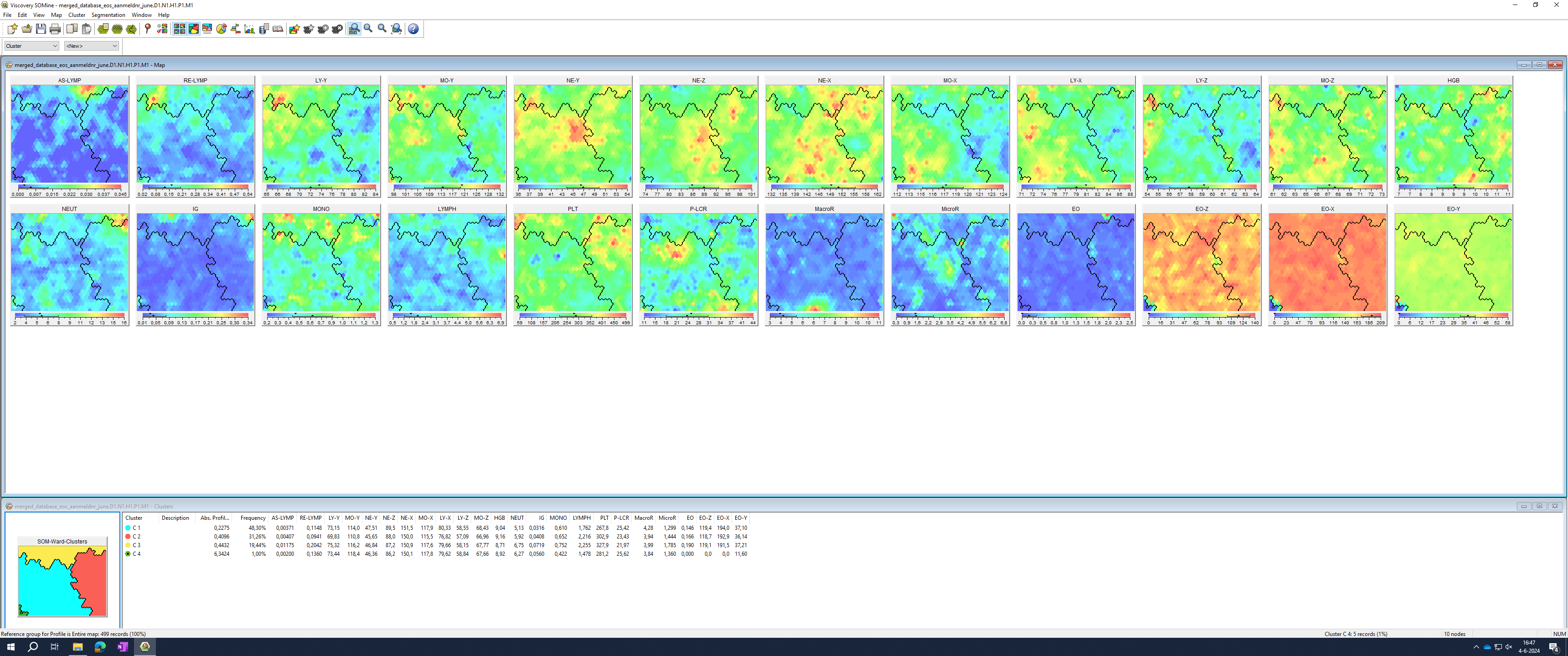

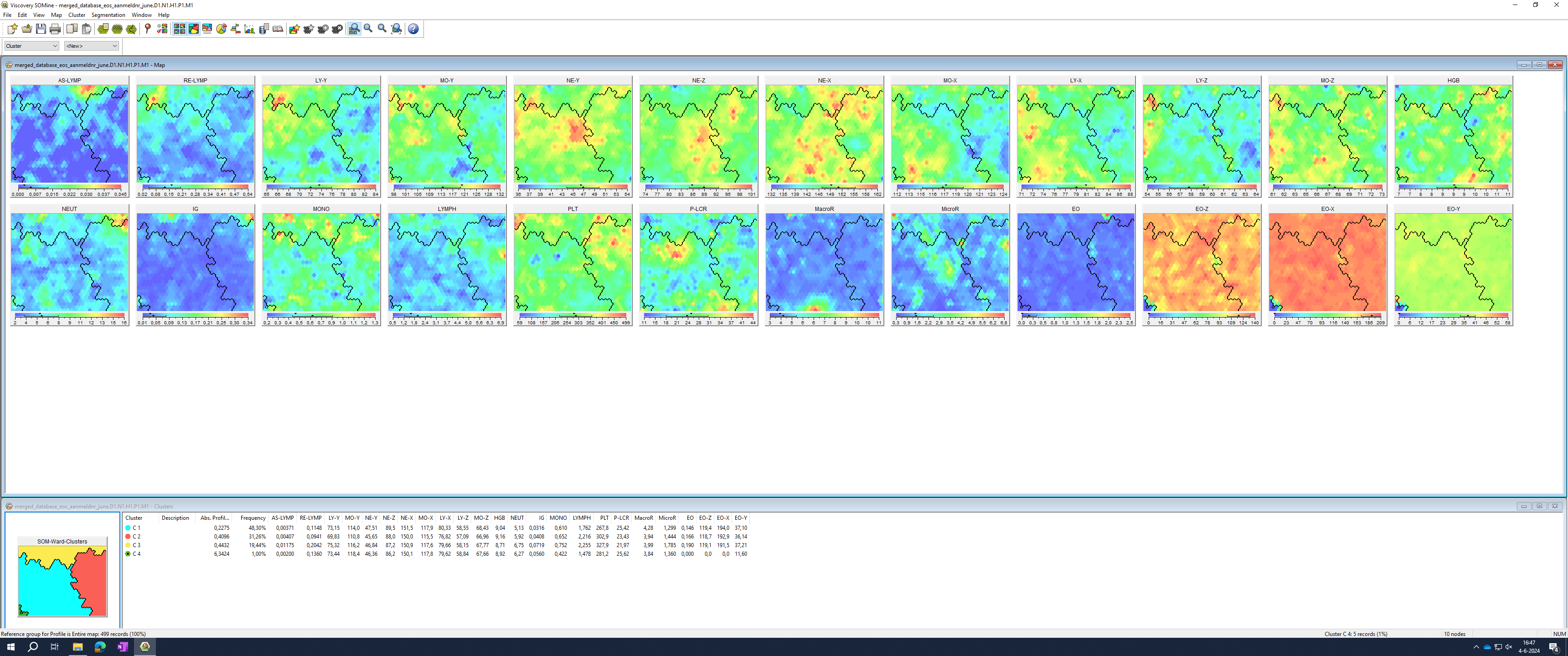


AS-LYMP

RE-LYMP

LY-Y

MO-Y

NEU-RI

NE-Z

NEUT

IG

MONO

LYMPH

PLT

P-LCR

NEUT-GI

MO-X

LY-X

LY-Z

MO-Z

HGB


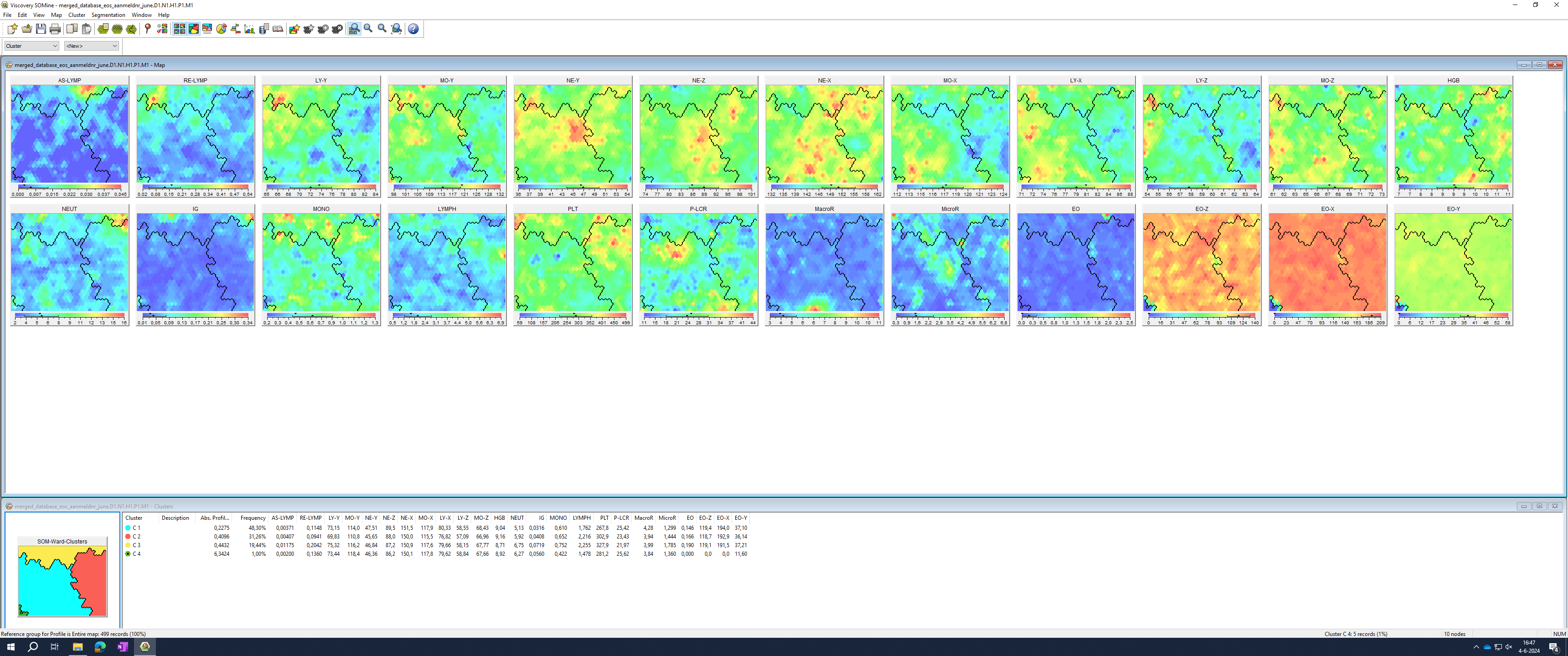


MacroR

MicroR

EO

EO-Z

EO-X

EO-Y

Supplementary Figure S1: Graphic presentation of the clusters. The Viscovery SOMline placed all patients on a specific position on the map. Subjects located close together resemble each other in terms of their cell population blood profile, and those with greater differences are positioned further apart.
